# Combination of inhibitors for two glycolytic enzymes portrays high synergistic efficacy against *Cryptosporidium parvum*

**DOI:** 10.1128/aac.00569-23

**Published:** 2023-09-01

**Authors:** Shahbaz M. Khan, Muhammad Rashid Bajwa, Rachael Y. Lahar, William H. Witola

**Affiliations:** 1 Department of Pathobiology, College of Veterinary Medicine, University of Illinois Urbana-Champaign, Urbana, Illinois, USA; The Children's Hospital of Philadelphia, Philadelphia, Pennsylvania, USA

**Keywords:** cryptosporidiosis, treatment, novel drug combinations, glycolysis molecular targets

## Abstract

*Cryptosporidium* is an intracellular protozoan parasite that causes serious enteric disease in humans and in a wide range of animals worldwide. Despite its high prevalence, no effective therapeutic drugs are available against life-threatening cryptosporidiosis in at-risk populations including malnourished children, immunocompromised patients, and neonatal calves. Thus, new efficacious drugs are urgently needed to treat all susceptible populations with cryptosporidiosis. Unlike other apicomplexans, *Cryptosporidium parvum* lacks the tricarboxylic acid cycle and the oxidative phosphorylation steps, making it solely dependent on glycolysis for metabolic energy production. We have previously reported that individual inhibitors of two unique glycolytic enzymes, the plant-like pyruvate kinase (CpPyK) and the bacterial-type lactate dehydrogenase (CpLDH), are effective against *C. parvum*, both *in vitro* and *in vivo*. Herein, we have derived combinations of CpPyK and CpLDH inhibitors with strong synergistic effects against the growth and survival of *C. parvum*, both *in vitro* and in an infection mouse model. In infected immunocompromised mice, compound combinations of NSC303244 + NSC158011 and NSC252172 + NSC158011 depicted enhanced efficacy against *C. parvum* reproduction and ameliorated intestinal lesions of cryptosporidiosis at doses fourfold lower than the total effective doses of individual compounds. Importantly, unlike individual compounds, NSC303244 + NSC158011 combination was effective in clearing the infection completely without relapse in immunocompromised mice. Collectively, our study has unveiled compound combinations that simultaneously block two essential catalytic steps for metabolic energy production in *C. parvum* to achieve improved efficacy against the parasite. These combinations are, therefore, lead compounds for the development of a new generation of efficacious anti-cryptosporidial drugs.

## INTRODUCTION

The opportunistic enteric parasite, *Cryptosporidium,* is a leading cause of waterborne and foodborne outbreaks of infectious diarrhea across the globe (1). In addition to diarrhea-related malnutrition, *Cryptosporidium* infection can result in reduced physical fitness and impaired cognitive function in early childhood (2). Of the currently recognized 44 *Cryptosporidium* species, the zoonotic *Cryptosporidium parvum* and the anthroponotic *Cryptosporidium hominis* are the most dominant species causing human infections (3). Several epidemiological studies conducted over the previous decade have reported *Cryptosporidium* to be an important cause of infectious diarrhea, growth defects, and mortality in young children living in under-developed countries (4 – 7). *Cryptosporidium* generally causes a self-limiting illness in immunocompetent individuals; however, those with reduced immunity such as malnourished children or HIV-infected patients are most at risk for severe disease. In the latter group of patients, especially the ones suffering from HIV/AIDS, *Cryptosporidium* infection is chronic, difficult to treat, and often life-threatening (8). Although the use of highly active antiretroviral therapy (ART) in HIV-infected patients has significantly reduced the global frequency and severity of cryptosporidiosis in this patient population (9), advanced AIDS patients with very low CD4+ cell counts continue to show high parasite infection rates despite receiving effective ART (10, 11). Furthermore, the use of ART in AIDS patients with chronic diarrhea has been associated with increased early mortality (12). Besides humans, *C. parvum* also causes severe diarrhea in neonatal animals, especially calves, leading to impaired growth rates and ensuing high economic and production losses (13).

Despite the strong impact of cryptosporidiosis on public health worldwide, effective control, treatment, and vaccination strategies are currently lacking (14). Several compounds have shown good efficacy in cell culture and laboratory animal-based models of cryptosporidiosis. A few among those have advanced to clinical trials in humans and agricultural animals but without success (14). Thus, there is a critical need for the development of novel anti-cryptosporidial therapeutics to ease the growing burden of cryptosporidiosis especially in low-income countries.

*C. parvum* seems to rely mainly on glycolysis for metabolic energy production due to the absence of functional mitochondria for the Krebs cycle and cytochrome-based electron transfer (15). Therefore, essential glycolytic enzymes such as *C. parvum* pyruvate kinase (CpPyK) and *C. parvum* lactate dehydrogenase (CpLDH) that are structurally and functionally unique to *C. parvum* are promising drug targets for this parasite (16, 17). Consistent with this notion, we have previously identified small-molecule inhibitors of recombinant CpPyK and CpLDH proteins’ enzymatic activities and demonstrated their efficacy against *C. parvum*, both *in vitro* and *in vivo* (18, 19). In *C. parvum*, glycolysis begins with the activation of glucose or fructose and ends with the production of pyruvate and ATP, the final reaction being catalyzed by CpPyK. Pyruvate is then rapidly converted to lactate (catalyzed by CpLDH) with the regeneration of cofactor NAD+, which is cycled back so that glycolysis can continue. Being cognizant of the importance of both CpPyK and CpLDH for efficient energy production in *Cryptosporidium*, we hypothesized that simultaneous inhibition of these enzymes could block glycolysis completely leading to severe growth and replication defects in the parasite. Thus, in the present study, we investigated the anti-cryptosporidial synergistic activities of CpPyK and CpLDH inhibitors in *C. parvum*-infected mammalian cells and immunocompromised mice. Using multiple approaches, we endeavored to determine specific combinations and concentration ratios of CpPyK and CpLDH inhibitors and tested them against *C. parvum* infection, both *in vitro* and *in vivo*, with the goal of deriving combinations with improved synergistic efficacy at relatively lower doses than those of the respective individual inhibitors.

## RESULTS

### Identification of non-toxic concentrations of compound combinations

The half-maximal cytotoxic concentration (CC_50_) values of CpPyK inhibitors (NSC234945, NSC252172, NSC303244, and NSC638080) and CpLDH inhibitors (NSC158011 and NSC10447) in human ileocecal colorectal adenocarcinoma (HCT-8) cell monolayers have been reported previously (18, 19). To evaluate compound combinations for *in vitro* anti-cryptosporidial effect, we first determined specific concentration ratios of combinations of CpPyK and CpLDH inhibitors that were not toxic to HCT-8 cells. We used a fixed-ratio ray design (20) to mix individual compounds using three mixture factor (*f*) values (0.25, 0.5, and 0.75) corresponding to three different ratios of the compounds based on their previously reported anti-cryptosporidial half-maximal effective concentration (EC_50_) values (18, 19). Use of multiple combination ratios for each combination allowed us to cover a larger spectrum of interaction between the two classes of compounds. Using this method, we derived a total of 24 unique compound mixtures of CpPyK + CpLDH inhibitors (Table 1). We used the WST-1 assay for evaluating the cytotoxicity of the compound mixtures against the host cell line (HCT-8) used for *in vitro* culture of *C. parvum*. The WST-1 assay is based on the reduction of tetrazolium salt to formazan within the mitochondria of metabolically active cells. Each compound mixture was analyzed at several increasing concentrations to determine its *in vitro* cytotoxicity against uninfected cells. The highest concentration of each compound mixture (Table 1; Table S1) that did not result in more than 20% mean percent toxicity (MPT) to host cells was used as the maximum concentration limit for subsequent *in vitro* efficacy assays.

**TABLE 1:** Combination of inhibitors of *C. parvum* pyruvate kinase (CpPyKi) and *C. parvum* lactate dehydrogenase (CpLDHi) according to the ray design model

Compound mixture (CpPyKi + CpLDHi)	Mixture factor (*f*)	Mix ratio	Highest concentration mix (μM:μM)
NSC234945 + NSC10447	0.25	1:1	100:100
	0.50	3:1	120:40
	0.75	9:1	135:15
NSC234945 + NSC158011	0.25	2:1	100:50
	0.50	6:1	120:20
	0.75	18:1	135:7.5
NSC252172 + NSC10447	0.25	1:6	5:30
	0.50	1:2	15:30
	0.75	3:2	30:20
NSC252172 + NSC158011	0.25	1:3	10:30
	0.50	1:1	30:30
	0.75	3:1	30:10
NSC303244 + NSC10447	0.25	1:6	5:30
	0.50	1:2	15:30
	0.75	3:2	30:20
NSC303244 + NSC158011	0.25	1:3	10:30
	0.50	1:1	30:30
	0.75	3:1	30:10
NSC638080 + NSC10447	0.25	1:6	5:30
	0.50	1:2	15:30
	0.75	3:2	30:20
NSC638080+NSC158011	0.25	1:3	10:30
	0.50	1:1	30:30
	0.75	3:1	30:10

### Synergistic *in vitro* anti-cryptosporidial efficacy of CpPyK and CpLDH inhibitors

**Fig 1:**
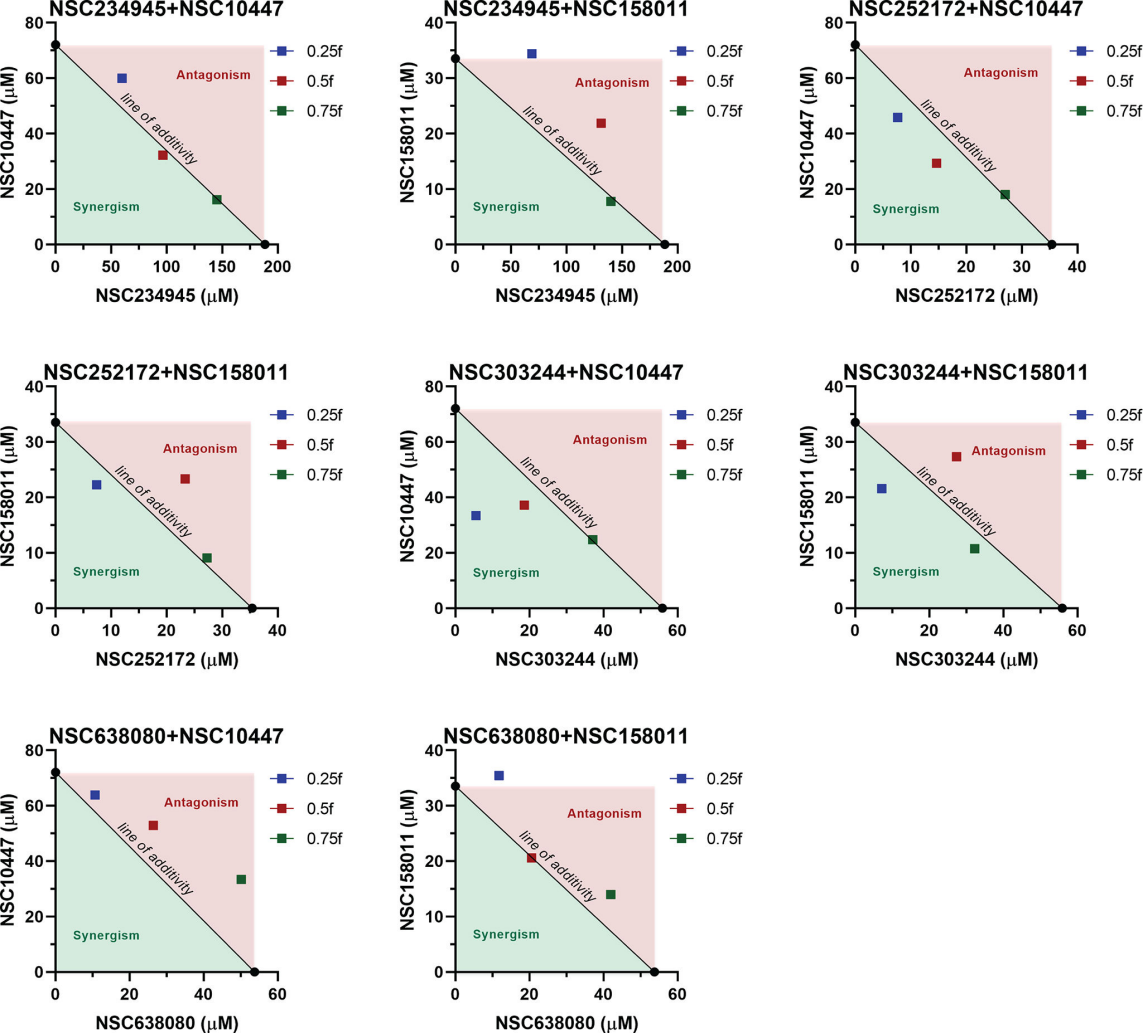
Isobolograms of multiple combination ratios of CpPyK and CpLDH inhibitors at the 75% parasite inhibition level. CpPyK and CpLDH inhibitors were combined in concentration ratios determined by three values of mixture factor (*f*) to prepare three different compound combinations (0.25*f*, 0.5*f*, and 0.75*f*). Concentration-response data of each compound mixture and individual compounds obtained from the *in vitro* parasite growth inhibition assays were analyzed by CompuSyn software to compute anti-*Cryptosporidium* EC_75_ values. The EC_75_ of CpPyK inhibitor plotted on the *x*-axis and the EC_75_ of CpLDH inhibitor plotted on the *y*-axis were joined to construct the line of additivity. Combination data points that fall below, on, or above the line of additivity indicate synergism, additivity, or antagonism, respectively. The data shown represent the mean of three independent experiments.

To ascertain the effect of combinations of CpPyK and CpLDH inhibitors on the growth and development of *C. parvum*, we performed *in vitro* parasite growth inhibition assays using infected HCT-8 cell monolayers. Increasing non-toxic concentrations of individual and mixture compounds were tested to evaluate their *in vitro* efficacy against *C. parvum*. The experimental data from test wells were normalized to that of wells treated with dimethyl sulfoxide (DMSO, negative control) and paromomycin (positive control). To demonstrate synergistic efficacy, several analytical methods such as isobologram analysis, combination index (CI) method, curve shift analysis, EC_50_ comparison, and dose-reduction index (DRI) analysis were utilized to analyze the concentration-response data. Figure 1 shows the isobologram plots of all the tested combinations. The isobologram approach is a graphical depiction of how drugs interact with each other from a pharmacological standpoint. Combination data points that fall below, on, or above the diagonal line formed by joining the individual compound EC_75_ values (line of additivity) indicate synergism, additivity, or antagonism, respectively. For the combinations of NSC252172 + NSC10447 and NSC303244 + NSC10447, concentration ratios of 1:6 (0.25*f*) and 1:2 (0.5*f*) were synergistic, while the 3:2 concentration ratio (0.75*f*) was additive (Fig. 1; Table 1). Likewise, combinations of NSC252172 + NSC158011 and NSC303244 + 158011 at 1:3 (0.25*f*) and 1:1 (0.5*f*) concentration ratios were synergistic and antagonistic, respectively (Fig. 1; Table 1). On the other hand, the 3:1 (0.75*f*) concentration ratio for NSC252172 + NSC158011 and NSC303244 + NSC10447 was antagonistic and synergistic, respectively (Fig. 1; Table 1). Interestingly, for all the above-mentioned combinations, compound mixtures with a mixture factor of 0.25*f* demonstrated the most synergy. The remaining four combinations (NSC234945 + NSC10447, NSC234945 + NSC158011, NSC638080 + NSC10447, and NSC638080 + NSC158011) were mostly antagonistic or additive at the tested combination ratios (Fig. 1; Table 1).

**Fig 2:**
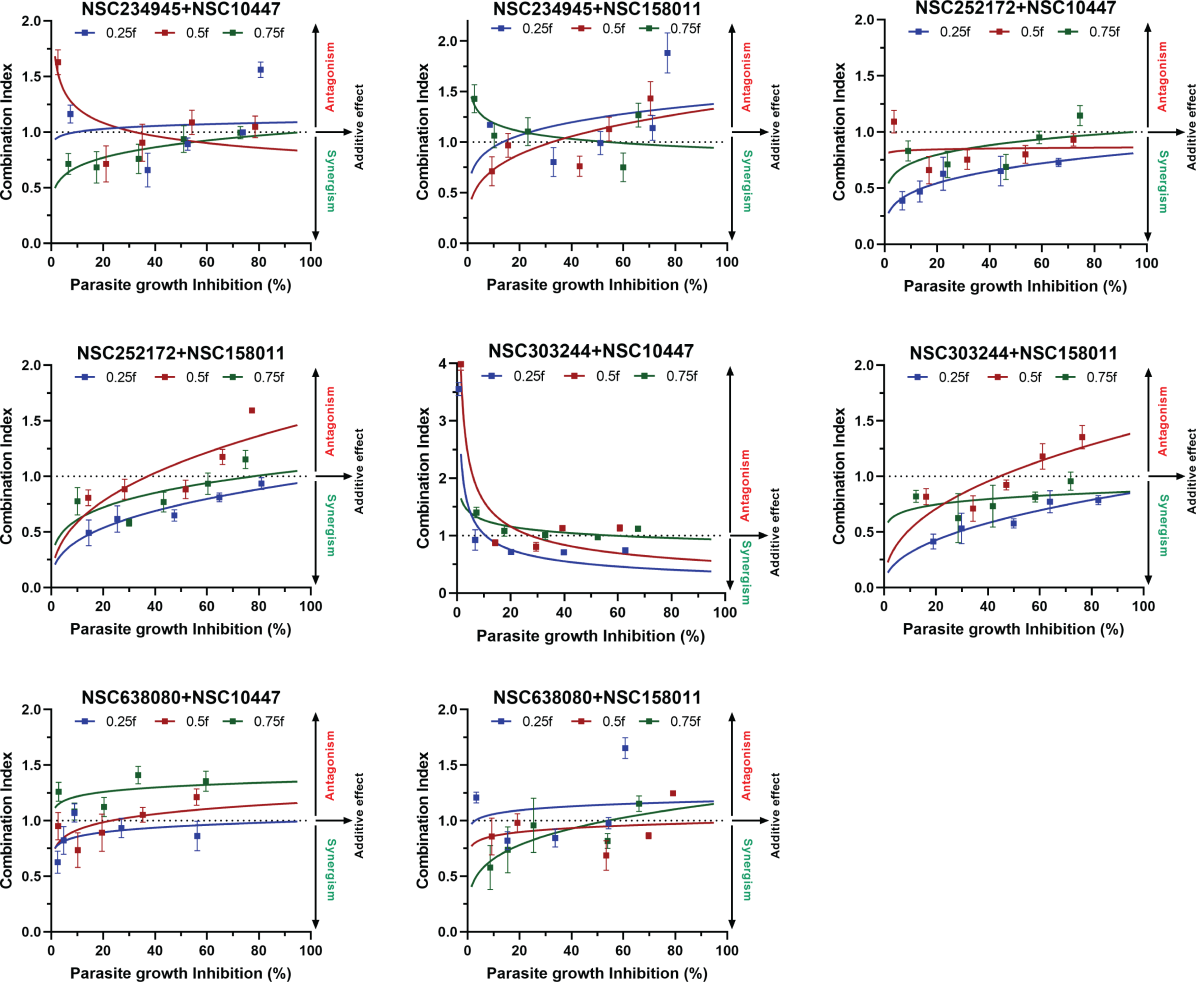
Combination index (CI) plots for mixtures of CpPyK and CpLDH inhibitors at different combination ratios. CpPyK and CpLDH inhibitors were mixed in concentration ratios determined by three values of mixture factor (*f*) to prepare three different compounds combinations (0.25*f*, 0.5*f*, and 0.75*f*). Concentration-response data points of each compound mixture acquired from the *in vitro C. parvum* growth inhibition assays were analyzed by CompuSyn software to calculate CI values. To construct CI plots, CI values of each compound mixture were plotted against the respective parasite growth inhibition levels. A CI of 1 (dotted line) indicates an additive effect between two agents, whereas values that fall below and above the line indicate synergism and antagonism, respectively. Data are presented as mean ± standard deviation of the mean (SD) of three independent experiments.

The synergistic activity of CpPyK- and CpLDH-inhibitor combinations was also reflected in the CI analyses of the *in vitro* concentration-response data (Fig. 2). Unlike the isobologram approach, the CI method offers a means of quantifying the pharmacologic interaction between two drugs. We used a computer software based on the CI algorithm of Chou and Talalay (21) to generate CI values for data from the parasite growth inhibition assays. In general, a CI value of 1 indicates an additive effect between two agents, whereas a CI value less than or greater than 1 indicates synergism or antagonism, respectively. The outcomes derived from the CI analyses were comparable to the results obtained from the isobologram analyses, and compounds combined in concentration ratios based on the 0.25*f* and 0.5*f* mixture factors showed the highest synergistic effects, particularly for NSC252172 + NSC10447 and NSC303244 + NSC10447 combinations (Fig. 2).

The CI and the isobologram analyses were well supported by the curve-shift analyses as evidenced by a left-ward shift of concentration-response curves for synergistic combinations and a right-ward shift for the antagonistic combinations, as compared to the corresponding single-compound curves (Fig. S1). This outcome meant that synergistic combinations required relatively lower concentration values compared with individual compounds to produce the same *in vitro* anti-cryptosporidial effect. Indeed, this was also evident when we compared the EC_50_ values of individual compounds and their combinations (Fig. S2). We found that synergistic combinations had lower EC_50_ values than their corresponding individual compounds and that the reverse was true for antagonistic combinations. For instance, the anti-cryptosporidial EC_50_ of the NSC303244 + NSC158011 (0.25*f*) combination was almost twofold lower than those of the individual compounds. On the other hand, the 0.75*f* NSC638080 + NSC10447 combination had a higher EC_50_ than both individual compounds (Fig. S2).

Furthermore, we calculated DRI values for compound mixtures of CpPyK and CpLDH inhibitors at three parasite inhibition levels using the CompuSyn software (ComboSyn, Inc.). The DRI method assesses how much the dosage of one or more compounds in the combination can be decreased while still producing similar effects compared to using the compounds individually. Drug combinations that demonstrate substantial reduction in dosage (DRI >1) can be recognized as those that act synergistically. Table S2 lists the DRI values of compound mixtures at the 50%, 75%, and 90% effect levels. DRI values were found to be greater than 1 for compounds in all combinations at all effect levels with higher overall DRI values for CpPyK inhibitors compared with CpLDH inhibitors.

### Anti-cryptosporidial efficacy of combinations of CpPyK and CpLDH inhibitors in infected mice

Based on the results derived from the synergy assessment methods described above, the combinations of NSC252172 + NSC158011, NSC252172 + NSC10447, and NSC303244 + NSC158011 prepared at the 0.25*f*, 0.5*f*, and 0.25*f* mixture factors, corresponding to the concentration ratios of 1:3, 1:2, and 1:3, respectively (Table 1), were found to possess the most anti-cryptosporidial potency and were, thus, selected for further investigation in an immunocompromised mouse model of cryptosporidiosis. Before the start of efficacy experiments, we tested synergistic CpPyK and CpLDH inhibitors individually and in combination at increasing doses in interferon gamma knockout (IFN-γ KO) mice to derive non-toxic doses for *in vivo* studies that did not induce any toxicity signs (changes in normal physical and mental activity, feeding pattern, body posture, body weight, fur condition, or occurrence of death) over 5 days of treatment (Table S3). Initially, the individual CpLDH inhibitors were tested at doses that were two- to fivefold lower than their reported effective doses (18) and were, thus, not expected to depict significant potency against *C. parvum* infection in mice. Additionally, individual CpPyK inhibitors were tested at doses lower than those used for CpLDH inhibitors since the former inhibitor class has generally been found to be more potent than the latter one in our previous study (19). The individually evaluated treatments were NSC252172 (CpPyK inhibitor) at 75 mg/kg, NSC303244 (CpPyK inhibitor) at 37.5 mg/kg, NSC158011 (CpLDH inhibitor) at 150 mg/kg, and NSC10447 (CpLDH inhibitor) at 200 mg/kg. Then, to determine whether administering the compounds in combination (CpLDH + CpPyK inhibitors) at lower doses would confer synergistic effects and, therefore, possess potency against *C. parvum*, the combinations were tested at doses that were at least 35% lower than their individual compound doses and at least fourfold lower than the combined reported effective doses of individual compounds (18, 19). Those evaluated combination treatments were NSC252172 + NSC158011 (100 mg/kg, 1:3 ratio), NSC252172 + NSC10447 (150 mg/kg, 1:2 ratio), and NSC303244 + NSC158011 (100 mg/kg, 1:3 ratio). Paromomycin was used as a positive control at 1,000 mg/kg once daily orally (22). Fecal oocyst shedding was confirmed by quantitative real-time PCR (qPCR) on day 3 post infection, following which oral gavage treatment of mice with compounds, paromomycin, or vehicle control commenced.

**Fig 3:**
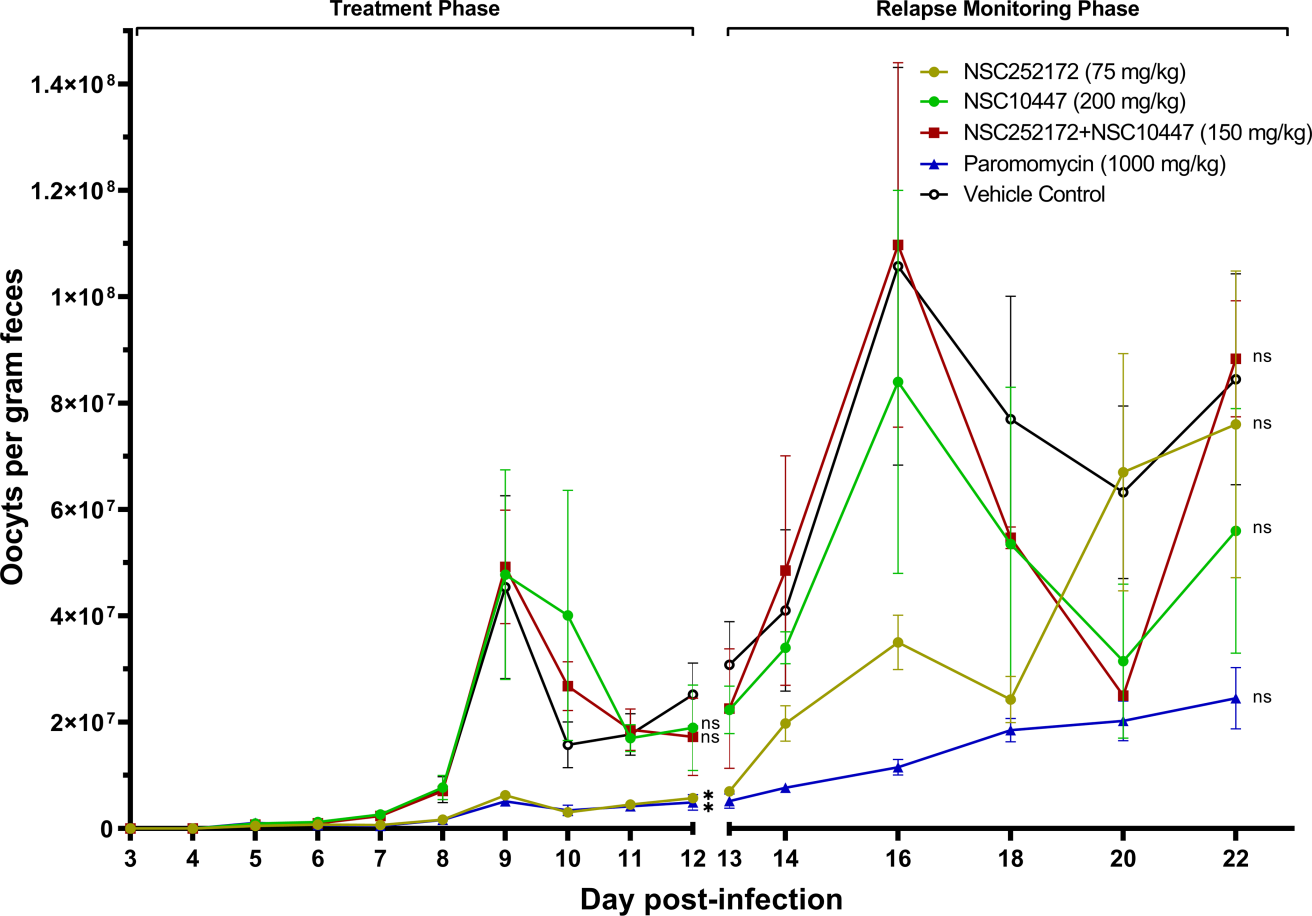
Analysis of the efficacy of NSC252172 and NSC10447 combination treatment against cryptosporidiosis. Male IFN-γ knockout mice were infected by oral gavage with 5 × 10^4^ *C. parvum* oocysts on day 0. Beginning at day 3 post infection, groups of mice were treated with NSC252172 (75 mg/kg), NSC10447 (200 mg/kg), NSC252172 + NSC10447 (150 mg/kg), paromomycin (1,000 mg/kg), or vehicle (100 µL of 25% DMSO in PEG 400), once daily until day 12. Oocyst shedding per gram of feces was determined by quantitative PCR on different days post infection. The data shown represent means for fecal samples from five mice (days 3–13 post infection) and three mice (days 14–22 post infection) per group. Bars represent standard error of the mean (SEM). On days 12 and 22 post infection, parasite load in different treatment groups was compared to the oocyst shedding in the vehicle control group by a non-parametric Kruskal-Wallis test with the Dunn’s multiple comparison test (ns, not significant; *P < 0.05).

As expected, vehicle control treatment group mice showed a progressive increase in oocysts shedding, with a peak load of about 5 × 10^7^ oocysts per gram of feces (OPG) being attained by day 9 post infection (Fig. 3). Treatments with individual compound NSC252172 (75 mg/kg) and paromomycin showed sustained lower (*P* < 0.05) oocysts counts than the vehicle control treatment until the end of treatment (Fig. 3). On the other hand, treatment with individual compound NSC10447 (200 mg/kg) had no notable effect on oocysts shedding when compared to the vehicle control treatment (Fig. 3). Furthermore, combination treatment with NSC252172 + NSC10447 (at lower combined dose than that of individual compounds) also did not have effect on oocysts shedding (Fig. 3), implying that this particular combination was not synergistic *in vivo*.

Post treatment, all groups of mice were observed for an additional 11 days for disease progression or relapse of infection. During this period, the average oocysts loads in the vehicle control-, the NSC252172 + NSC10447 combination-, and the individual NSC10447-treatment groups rose to higher peaks at day 16 post infection than those observed earlier at day 9 post infection (Fig. 3). Interestingly, the oocysts loads in the NSC252172- and paromomycin-treatment groups (that had earlier been lower than in the vehicle control group) had started to rebound (Fig. 3). This indicated that the *C. parvum* infection in mice treated with NSC252172 or paromomycin relapsed promptly after treatment withdrawal, suggesting that these compounds inhibited parasite reproduction without killing the parasite.

**Fig 4:**
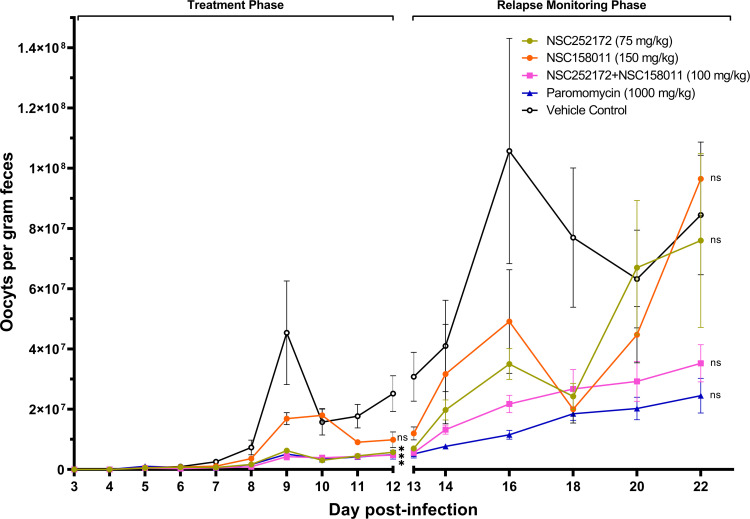
Analysis of the efficacy of NSC252172 and NSC158011 combination treatment against cryptosporidiosis. Male IFN-γ knockout mice were infected by oral gavage with 5 × 10^4^ *C. parvum* oocysts on day 0. Beginning at day 3 post infection, groups of mice were treated with NSC252172 (75 mg/kg), NSC158011 (150 mg/kg), NSC252172 + NSC158011 (100 mg/kg), paromomycin (1,000 mg/kg), or vehicle (100 µL of 25% DMSO in PEG 400), once daily until day 12. Oocyst shedding per gram of feces was determined by quantitative PCR on different days post infection. The data shown represent means for fecal samples from five mice (days 3–13 post infection) and three mice (days 14–22 post infection) per group. Bars represent standard error of the mean. On days 12 and 22 post infection, parasite load in different treatment groups was compared to the oocyst shedding in the vehicle control group by a non-parametric Kruskal-Wallis test with the Dunn’s multiple comparison test (ns, not significant; *P < 0.05).

Next, we tested the effect of NSC252172 + NSC158011 combination in comparison to the individual compounds. Treatment of mice with NSC158011 (150 mg/kg) resulted in lowered oocysts load in feces of infected mice at the end of the treatment period, although not statistically significant (*P* > 0.05), when compared to the vehicle control group (Fig. 4). Notably, NSC252172 + NSC158011 combination treatment and individual NSC25172 and paromomycin treatments resulted in significant (*P* < 0.05) reductions in oocysts loads starting at day 9 until the end of treatment (Fig. 4). But as earlier observed for paromomycin and NSC158011, cessation of treatment with NSC252172 + NSC158011 combination or individual NSC158011 led to a relapse of infection (Fig. 4).

**Fig 5:**
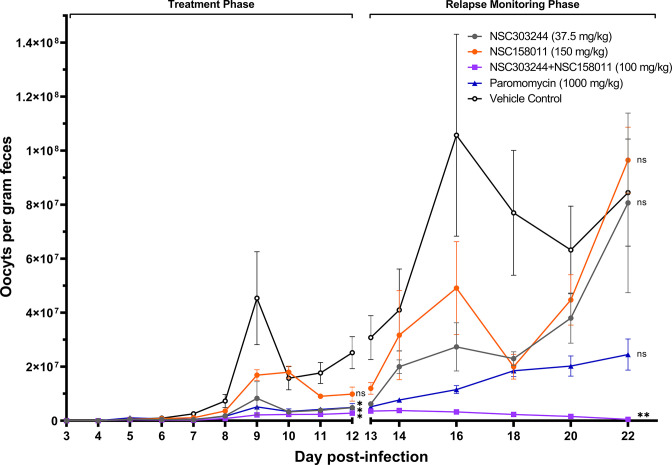
Analysis of the efficacy of NSC303244 and NSC158011 combination treatment against cryptosporidiosis. Male IFN-γ knockout mice were infected by oral gavage with 5 × 10^4^ *C. parvum* oocysts on day 0. Beginning at day 3 post infection, groups of mice were treated with NSC303244 (37.5 mg/kg), NSC158011 (150 mg/kg), NSC303244 + NSC158011 (100 mg/kg), paromomycin (1,000 mg/kg), or vehicle (100 µL of 25% DMSO in PEG 400), once daily until day 12. Oocyst shedding per gram of feces was determined by quantitative PCR on different days post infection. The data shown represent means for fecal samples from five mice (days 3–13 post infection) and three mice (days 14–22 post infection) per group. Bars represent standard error of the mean. On days 12 and 22 post infection, parasite load in different treatment groups was compared to the oocyst shedding in the vehicle control group by a non-parametric Kruskal-Wallis test with the Dunn’s multiple comparison test (ns, not significant; *P < 0.05; **P < 0.01).

In the third treatment trial, we compared the effects of combination of NSC303244 + NSC158011 to the respective individual compounds, paromomycin, and vehicle control treatments on oocysts load in *C. parvum*-infected mice. In a similar manner to individual treatment with NSC158011, treatment with individual NSC303244 led to reduced oocysts loads that relapsed after treatment was withdrawn (Fig. 5). Intriguingly, NSC303244 + NSC158011 combination treatment resulted in sustained very low oocysts counts in feces that continued to decline even after cessation of treatment, reaching nearly zero count by day 22 post infection when the oocysts loads in other treatment groups were rapidly increasingly exponentially (Fig. 5). This strongly indicated that combination of NSC303244 (CpPyK inhibitor) and NSC158011 (CpLDH inhibitor) resulted in a synergistic effect that was parasiticidal. Corroboratively, when the entire shedding-monitoring period was considered, the combination of NSC303244 and NSC158011 was the only treatment observed to significantly decrease (*P* < 0.01) the average fecal oocysts count in infected mice in comparison to the vehicle control (Table 2).

**TABLE 2:** Average counts of oocysts shedding per gram of feces in infected mice from day 3 to day 22 post infection as determined by qPCR quantification

Treatment group	Average oocyst count per gram feces
NSC252172 (75 mg/kg)	15,766,647^*a*^
NSC303244 (37.5 mg/kg)	13,674,439^*a*^
NSC158011 (150 mg/kg)	19,632,226^*a*^
NSC10447 (200 mg/kg)	26,110,916^*a*^
NSC252172 + NSC158011 (100 mg/kg)	9,466,084^*a*^
NSC303244 + NSC158011 (100 mg/kg)	1,664,310^*b*^
NSC252172 + NSC10447 (150 mg/kg)	29,490,632^*a*^
Paromomycin (1,000 mg/kg)	6,832,361^*a*^
Vehicle control	32,364,839

^*a*^ Not significantly different from vehicle control.

^*b*^ *P* < 0.01 by a non-parametric Kruskal-Wallis test with the Dunn’s test for multiple comparisons between the treatments and the vehicle control.

**Fig 6:**
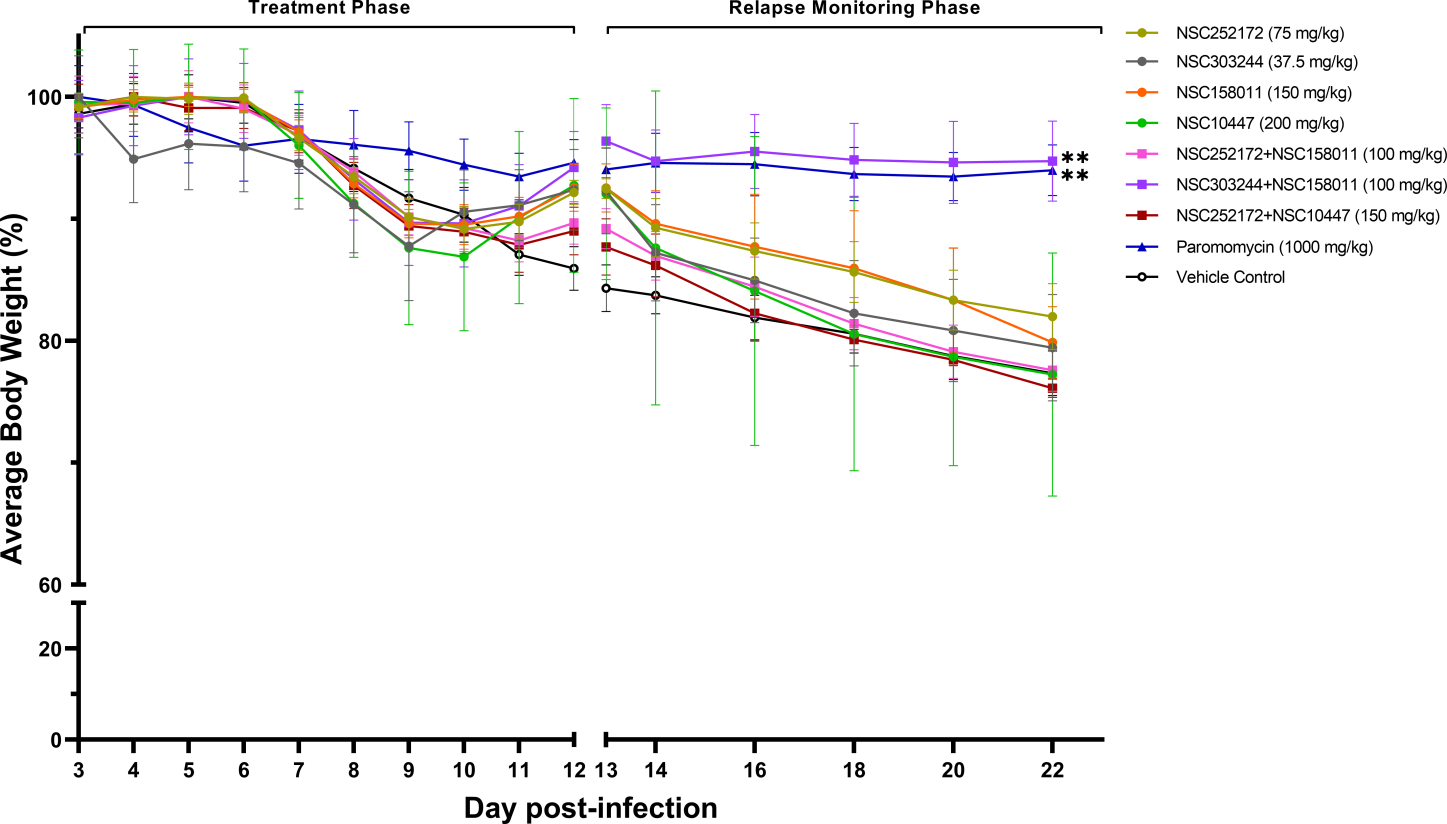
Determination of average body weight of mice during and after treatment against cryptosporidiosis. Male IFN-γ knockout mice were infected by oral gavage with 5 × 10^4^ *C. parvum* oocysts on day 0. Beginning day 3 post infection, groups of mice were treated with CpPyK and CpLDH inhibitors either individually or in combination at the indicated doses, paromomycin (1,000 mg/kg) or vehicle (100 µL of 25% DMSO in PEG 400), once daily until day 12. Body weight was recorded daily during and after the treatment period and is shown on the *y*-axis representing the average body weight of each treatment group normalized to the respective mean body weight on day 3. The data shown represent mean body weights from five mice (days 3–13 post infection) and three mice (days 14–22 post infection) per group. Bars represent standard error of the mean. On day 22 post infection, mean body weights of various treatment groups were compared to that of the vehicle control group by a parametric one-way analysis of variance (ANOVA) test with the Dunnett’s multiple comparison test (**P < 0.01).

Analysis of the daily changes in the body weight of mice showed that the mean weight of the untreated mice decreased by 22% (from 24.3 g on day 3 to 19 g on day 22 post infection) (Fig. 6). In contrast, the average weight of the mice treated with the NSC303244 + NSC158011 combination only dropped by 3% (from 24.4 g on day 3 to 23.6 g on day 22 post infection), a nearly sevenfold difference compared to the vehicle control group (*P* < 0.01) (Fig. 6). Interestingly, paromomycin-treated mice despite shedding considerable numbers of oocysts during the monitoring phase showed no apparent clinical signs of infection and minimal loss of body weight from day 3 until day 22 (1.5 g, ~6%). Griffiths et al. reported a similar finding after treating *C. parvum*-infected IFN-γ KO mice with a lower dose of paromomycin (22). All other treatment groups lost body weight compared to the untreated group of mice by the end of the experiment (Fig. 6).

**Fig 7:**
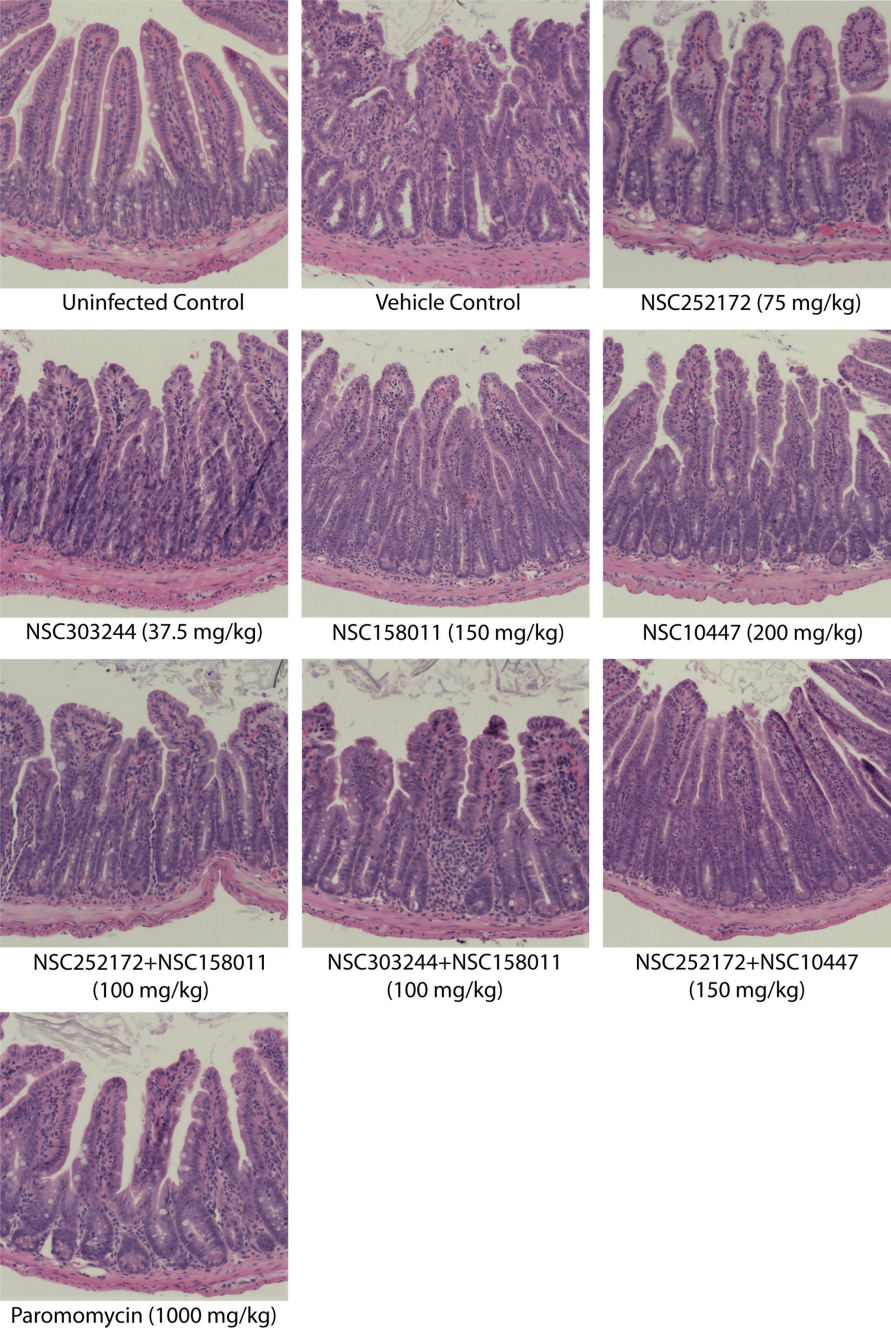
Hematoxylin- and eosin-stained histological sections from the distal small intestines of *C. parvum*-infected mice with or without treatment. Male IFN-γ knockout mice were infected by oral gavage with 5 × 10^4^ *C. parvum* oocysts on day 0. Beginning day 3 post infection, groups of mice were treated with NSC272172 (75 mg/kg), NSC303244 (37.5 mg/kg), NSC158011 (150 mg/kg), NSC10447 (200 mg/kg), NSC252172 + NSC158011 (100 mg/kg), NSC303244 + NSC158011 (100 mg/kg), NSC252172 + NSC10447 (150 mg/kg), paromomycin (1,000 mg/kg) as positive control, or vehicle (100 µL of 25% DMSO in PEG 400), once daily until day 12. The uninfected control group of mice was administered an equivalent volume of the compound vehicle. On day 13 post infection, randomly selected mice were sacrificed, and the distal small intestines were processed for histology and stained with hematoxylin and eosin. Representative microscopy images of samples harvested from at least two mice per treatment group on day 13 after infection are shown here.

One day after cessation of treatment, two mice per treatment group were randomly selected and sacrificed for intestinal histopathological examination. As expected, the uninfected control group samples showed a healthy intestinal mucosa with prominent villi. There were no obvious differences in the structure and organization of the intestinal mucosa of mice that were treated with NSC252172, NSC303244, NSC252172 + NSC158011, NSC303244 + NSC158011, or the positive control paromomycin when compared to the uninfected mice (Fig. 7). These findings were consistent with the significantly reduced number of oocysts detected in these groups during the treatment phase. On the contrary, mice treated with the vehicle control had lesions characterized by mucosal erosion, villous atrophy, hypertrophy of the crypts, and inflammation. Similar but milder lesions were observed in the intestinal mucosa of mice treated with NSC158011, NSC10447, and the NSC252172 + NSC10447 combination (Fig. 7).

**Fig 8:**
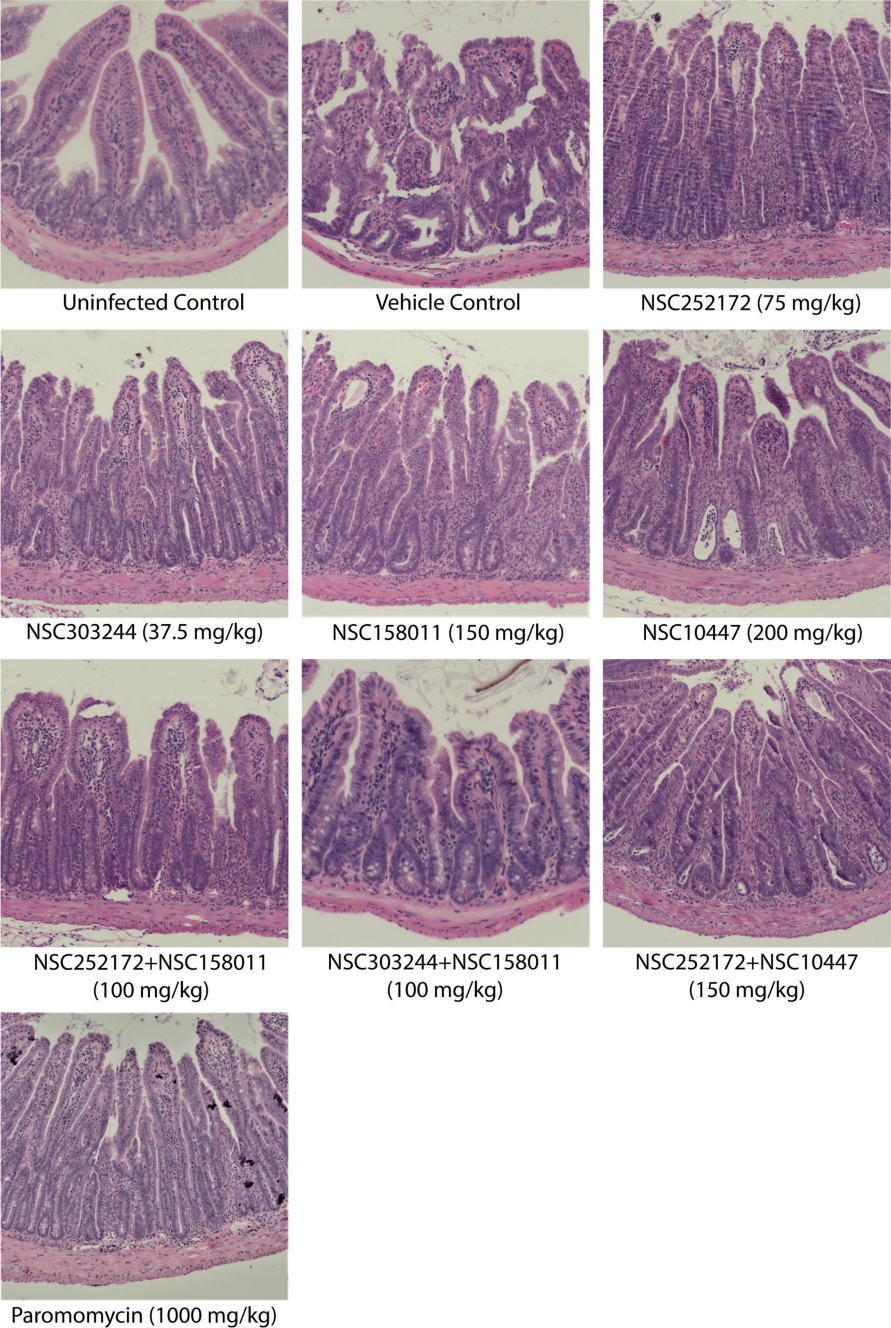
Hematoxylin- and eosin-stained histological sections from the distal small intestines of *C. parvum*-infected mice with or without treatment. Male IFN-γ knockout mice were infected by oral gavage with 5 × 10^4^ *C. parvum* oocysts on day 0. Beginning day 3 post infection, groups of mice were treated with NSC272172 (75 mg/kg), NSC303244 (37.5 mg/kg), NSC158011 (150 mg/kg), NSC10447 (200 mg/kg), NSC252172 + NSC158011 (100 mg/kg), NSC303244 + NSC158011 (100 mg/kg), NSC252172 + NSC10447 (150 mg/kg), paromomycin (1,000 mg/kg) as positive control, or vehicle (100 µL of 25% DMSO in PEG 400), once daily until day 12. The uninfected control group of mice was administered an equivalent volume of the compound vehicle. On day 23 post infection, randomly selected mice were sacrificed, and the distal small intestines were processed for histology and stained with hematoxylin and eosin. Representative microscopy images of samples harvested from at least two mice per treatment group on day 23 after infection are shown here.

To evaluate the impact of treatment discontinuation on the recurrence or advancement of the disease, we sacrificed all the remaining mice on day 23 and performed histopathological analysis of intestinal tissue samples. As anticipated, at this stage, the vehicle control mice depicted pronounced pathological lesions of severe illness including denudation and blunting of intestinal villi, severe mucosal erosion, and marked widespread infiltration of inflammatory cells (Fig. 8). Similar to the vehicle control, mice from the NSC158011, NSC10447, and NSC252172 + NSC10447 treatment groups showed an escalation of pathological changes in the small-intestinal mucosa by day 23 (Fig. 8). Likewise, on day 23, we observed microscopic intestinal lesions of cryptosporidiosis in mice treated with NSC252172, NSC303244, NSC252172 + NSC158011, or paromomycin, suggesting a relapse of infection, which correlated with the elevated oocyst load observed after treatment was discontinued on day 12. However, these lesions were milder than the ones observed in the vehicle control on that day. In contrast, the NSC303244 + NSC158011 combination had a noticeable effect in preventing intestinal pathology in infected mice even after treatment cessation, as evidenced by the preservation of the intestinal epithelium with intact villi on day 23 (Fig. 8).

## DISCUSSION

Cryptosporidiosis is a leading cause of diarrheal illness worldwide. Yet, the development of efficacious therapies for this important parasitic infection remains a significant challenge in public health. Currently, there are no effective treatments for cryptosporidiosis, especially in vulnerable populations including children, immunocompromised individuals, and neonatal calves. Nitazoxanide is the only drug approved by FDA for treating cryptosporidiosis in immunocompetent humans, but it remains ineffective for those who are susceptible to the disease (23). Although halofuginone lactate is licensed for treating calf cryptosporidiosis in Europe and Canada, it is not approved for use in the United States. Regardless, halofuginone lactate has a limited safety margin and is contraindicated in dehydrated animals with diarrhea, a clinical finding consistent with cryptosporidiosis in neonatal calves (24). Thus, finding effective treatments for this human and animal disease continues to be a top priority. We provide compelling evidence for the effectiveness of combination therapy in the treatment of cryptosporidiosis by deriving specific combinations of compounds that target two unique enzymes in the glycolytic pathway for the generation of metabolic energy for the parasite, leading to greater efficacy than individual compounds in cell culture and animal models at reduced doses.

*C. parvum* lacks functional mitochondria along with the genes encoding enzymes of the Krebs cycle and the electron transport chain (15). Consequently, the glycolytic pathway is a critical metabolic pathway in this parasite and is believed to be essential for parasite survival and pathogenesis. By simultaneously targeting two enzymes in this pathway, we aimed to completely disrupt the energy production in the parasite and eventually kill it. While glycolysis is a conserved metabolic pathway in most eukaryotic organisms, making it challenging to develop drugs that selectively target the parasite without affecting the host, the cryptosporidial glycolytic enzymes are, nevertheless, significantly different from those found in mammals. Further, our previous studies have demonstrated that inhibitors against two of these enzymes, CpPyK and CpLDH, possess high therapeutic indices for treating experimental *C. parvum* infections (18, 19). Therefore, in the present study, we tested the efficacy of various combinations of CpPyK and CpLDH inhibitors against *C. parvum* in cell culture and animal models. Our results showed that CpPyK and CpLDH inhibitors, when combined at suboptimal concentrations, were able to effectively inhibit the growth and proliferation of these parasites compared to the use of either of the compounds alone. Of particular significance, the combination of NSC303244 (CpPyK inhibitor) and NSC158011 (CpLDH inhibitor) was successful in preventing relapse of infection in IFN-γ knockout mice, a suitable and widely used immunodeficient model of cryptosporidiosis (22), even after discontinuation of treatment. This is a promising finding as relapse is a common problem in the treatment of cryptosporidiosis in susceptible individuals, and management of the disease often requires extended treatment periods or multiple rounds of treatment (25, 26).

Most drug discovery efforts for cryptosporidiosis have historically relied on testing individual compounds, and none of these studies has been able to translate the research findings into an effective cure for cryptosporidiosis to date (27). Some *Cryptosporidium* drug discovery efforts, however, have screened a combination of two or more drugs and noted a marked improvement in efficacy against the parasite in cell culture (28 – 30). Moreover, several combination therapies have been shown to improve clinical outcomes compared with monotherapy in immunocompromised humans and animals with *Cryptosporidium* infections (14). However, these potential anti-cryptosporidial therapies have not been followed up in large controlled clinical trials despite the increasing importance of combination therapies for the treatment of other related apicomplexan protozoan infections including *Plasmodium*, *Babesia,* and *Toxoplasma*.

Combination therapy has several advantages over monotherapy, including increased efficacy, reduced toxicity, and decreased risk of drug resistance. The effectiveness of the combination therapy in our study can be attributed to the fact that targeting two important enzymes in the glycolytic pathway effectively disrupts the generation of metabolic energy for the parasite. CpPyK plays a central role in the energy metabolism of *C. parvum* and serves as a key metabolic control point as both the substrate (phosphoenolpyruvate) and the product (pyruvate) of this enzyme feed into several metabolic pathways essential to the parasite. Similarly, CpLDH plays a critical role in glycolysis, allowing for the interconversion of pyruvate and lactate and the regeneration of NAD+ from NADH which is required for glycolysis to continue. In *C. parvum*, CpPyK is constitutively expressed in the cytosol during all parasite stages (31) with relatively higher expression seen in intracellular stages than oocysts and sporozoites (32, 33), suggesting that this enzyme is vital for energy production during intracellular parasite growth. In contrast, CpLDH is localized mainly in the cytosol in extracellular parasites including sporozoites within oocysts, free sporozoites, and merozoites but becomes associated with the parasitophorous vacuole membrane (PVM) during intracellular development, suggesting involvement of the PVM in the energy metabolism of *C. parvum* (34). Moreover, CpLDH expression in intracellular parasites is significantly weaker than that observed in extracellular parasite stages (32, 33), indicating that this enzyme is mainly utilized for energy metabolism in the invading life cycle stages. Concurrent inhibition of these enzymes, therefore, produces strong synergistic inhibitory effects on the energy metabolism of all parasite stages by shutting down ATP generation through the glycolytic cycle. Our findings are significant in light of the worrying emergence of drug-resistant parasites during anti-cryptosporidial treatment as recently evidenced using a promising tRNA synthetase inhibitor (35).

The effectiveness of drug combinations may vary depending on the individual properties of each drug, their dosages, drug ratios, and potential drug-drug interactions. For some classes of compounds, certain ratios may have a synergistic effect, while others might be antagonistic, a widely acknowledged phenomenon in pharmacology (36). Thus, when combining drugs, multiple ratios should be used to achieve the desired therapeutic effect while minimizing side effects. In this study, we used a fixed-ratio design based on individual EC_50_ values for combining CpPyK and CpLDH inhibitors. Moreover, we used three values of the mixture factor to design different compound mixtures for each combination and assessed synergy by multiple approaches. By using multiple fixed ratios, we were able to explore different combinations and concentrations of compounds and test them independently for both cytotoxicity and anti-cryptosporidial efficacy in mammalian cells. We observed that different concentration-ratio mixtures within the same combination showed markedly different responses to each other. Importantly, the various synergy-assessment methods used in our study demonstrated this crucial outcome, with the ability to identify effective and safe compound mixtures with the greatest synergistic interaction for *in vivo* studies.

The NSC251272 + NSC10447 combination, although found to be synergistic *in vitro*, showed reduced efficacy in infected mice. There are several possible reasons to explain this unexpected outcome. For example, the pharmacokinetics of the individual drugs in this combination may differ, and their levels and distribution *in vivo* could be impacted differently, leading to a reduced effect compared to *in vitro*. Other factors that may be responsible for reduced *in vivo* efficacy include drug-drug interactions and differences in cellular environment at the target site. Meanwhile, based on the monitoring of relapse of infection in infected mice, the NSC303244 + NSC158011 combination was apparently parasiticidal for *C. parvum,* while most individual compounds, paromomycin, and the combination of NSC252172 and NSC158011 were parasitistatic. A “cidal” antiparasitic agent is one that kills parasites without depending on the patient’s immune system for assistance. On the other hand, a “static” agent is one that inhibits the growth and multiplication of the parasite without necessarily killing it, thus allowing the patient’s immune system to kill off the parasite leading to recovery from the infection. Clearly, parasiticidal agents are more desirable for treating cryptosporidiosis in malnourished children, immunocompromised humans, and neonatal calves, a patient population in which there is minimal host defense. In addition, the development of such drugs is essential in the fight against the inevitable rise of resistance to anti-cryptosporidial drugs. Thus, the parasiticidal nature of the NSC303244 + NSC158011 combination is extremely valuable for our efforts to find a much-needed cure for cryptosporidiosis.

## MATERIALS AND METHODS

### Parasites and host cells

The *C. parvum* AUCP-1 isolate was maintained and propagated in male Holstein calves. *C. parvum* oocysts were extracted and purified from freshly collected calf feces by sequential sieve filtration, Sheather’s sugar flotation, and discontinuous sucrose density gradient centrifugation (37, 38). Purified oocysts were washed and stored in phosphate-buffered saline (PBS) at 4°C and used within 3 months to ensure maximum viability as judged by excystation. *C. parvum* sporozoites were excysted from oocysts as previously described (39) but with slight modifications. Briefly, 1 × 10^8^ purified *C. parvum* oocysts were suspended in 500 µL of PBS and treated with an equal volume of 40% commercial laundry bleach for 10 min on ice, followed by four washes in PBS containing 1% bovine serum albumin. Oocysts were suspended in Hanks balanced salt solution (HBSS; Corning), incubated for 60 min at 37°C, and mixed with an equal volume of warm 1.5% sodium taurocholate in HBSS, followed by further incubation for 60 min at 37°C. The excysted sporozoites were pelleted by centrifugation and resuspended in RPMI-1640 medium containing 1% heat-inactivated fetal bovine serum (Hi-FBS; Gibco). The sporozoites were separated from oocyst shells and unexcysted oocysts by passing the suspension through a sterile 5-µm syringe filter (Millex; Millipore). Purified sporozoites were enumerated with a hemocytometer and used immediately for infection of cell monolayers.

For *in vitro* studies, HCT-8 cells (HCT-8 [HRT-18]; ATCC CCL-244, RRID:CVCL_2478) were used. Cells were cultured and maintained in RPMI-1640 medium (Gibco) supplemented with 2.5 g/L of glucose, 1 mM sodium pyruvate, 1.5 g/L of sodium bicarbonate, 10% Hi-FBS (Gibco), and 1 × antibiotic-antimycotic (Gibco) at 37°C with 5% CO_2_ in a humidified incubator.

### Chemical compounds

Compounds, originally from the National Cancer Institute Diversity Set VI chemical library, were procured from different chemical vendors: NSC234945 and NSC10447 were obtained from A2B Chem LLC, USA; NSC158011 and NSC252172 were acquired from AKos Consulting & Solutions GmbH, Germany; and NSC303244 and NSC638080 were purchased from ChemSpace LLC, USA. All compounds were ≥95% pure as determined by liquid chromatography-mass spectrometry and/or proton nuclear magnetic resonance analysis and were shipped as powder. Upon receipt, compounds were individually reconstituted in cell culture grade DMSO (ATCC) and stored as stock solutions at −20°C. For *in vitro* experiments, stock solutions of compounds and their combinations were diluted in RPMI-1640 medium to produce working solutions for testing cytotoxicity and anti-cryptosporidial efficacy. For *in vivo* studies, stock solutions of individual compounds and their combinations were diluted in 100% (vol/vol) polyethylene glycol 400 (PEG 400; Rigaku Reagents) to a final DMSO concentration of 25%. Paromomycin sulfate (Thermo Scientific) was used as a control for *in vitro* and *in vivo* studies because of its previously reported activity against *C. parvum* (22, 40).

### Compound combination model

The fixed -ratio or ray design model of drug combination (20) was used for combination of compounds for *in vitro* studies. Briefly, two classes of compounds (CpPyK and CpLDH inhibitors) were combined to prepare a compound mixture *Z* according to the formula:


Z=fA+(1−f)B,

where A and B are *in vitro* anti-cryptosporidial half-maximal effective concentration (EC_50_) values of individual CpPyK and CpLDH inhibitors, respectively, and *f* is the mixture factor. Compounds were combined in concentration ratios based on three values of *f* (0.25, 0.5, and 0.75) to prepare three compound mixtures for each compound combination. Each compound mixture was considered a new compound and tested *in vitro* for cytotoxicity and anti-*Cryptosporidium* efficacy.

### *In vitro* cytotoxicity assay

A colorimetric assay using the cell proliferation reagent WST-1 (Roche) was performed for the quantification of *in vitro* cytotoxicity of compound combinations in HCT-8 cells. About 5 × 10^4^ HCT-8 cells were seeded per well in flat-bottomed 96-well plates and grown overnight in 200 µL of supplemented RPMI-1640 medium. Upon reaching 80%–90% confluency, cells were treated in quadruplicate with increasing concentrations of each fixed-ratio chemical compound mixture (reconstituted in DMSO) for 48 h. The volume of DMSO did not exceed 1% of the total culture volume in any of the wells to avoid DMSO toxicity to the cells. Control wells received equivalent volumes of DMSO instead of the compounds. Ten microliters of the WST-1 reagent were added to each well after 48 h of culture, and the plates were incubated for 30 min at 37°C with 5% CO_2_ under dark conditions. Following incubation, the plates were shaken thoroughly, and 150 µL of the medium from each well was transferred to a new clear flat-bottomed black 96-well plate (Corning). Absorbance was read at a test wavelength of 440 nm and a reference wavelength of 690 nm using a multi-mode microplate reader (Spectra Max iD5; Molecular Devices). Mean percent toxicity of each compound mixture was derived by dividing the difference in absorbance between the compound-treated cells and the DMSO-treated cells by the absorbance from the DMSO-treated cells and multiplying the product by 100:


$$MPT=[(Mean\;OD_{DMSO-treated}-Mean\;OD_{compound-treated})÷\;\;Mean\;OD_{DMSO-treated}]\times 100$$

where

- MPT is the mean percent toxicity of compound.
- Mean OD_DMSO-treated_ is the average absorbance value of quadruplicate wells treated with volumes of DMSO equivalent to that of the compound-treated wells.
- Mean OD_compound-treated_ is the average absorbance value of quadruplicate wells treated with the compound.

### *In vitro Cryptosporidium* growth inhibition assay

HCT-8 cells were seeded and grown to confluency in supplemented RPMI-1640 medium in flat-bottomed 96-well plates. After replacing the old medium with fresh RPMI-1640 medium containing 1% Hi-FBS (infection medium), plates were inoculated with 10^5^ freshly excysted *C. parvum* sporozoites per well. Immediately after infection, HCT-8 monolayers were treated with twofold dilution series of the highest sub-toxic (<20% MPT) concentration of individual compounds and compound mixtures. Control infected cells were treated with volumes of DMSO (up to a maximum final concentration of 1%) equivalent to those used for the compound-treated cultures. Paromomycin reconstituted in sterile distilled water was added to a separate set of wells as a positive control at a final concentration of 400 µM. Control wells with uninfected monolayers were also included for background subtraction. After 48 h of culture at 37°C with 5% CO_2_, the cultures were analyzed for parasite infectivity and proliferation by a direct immunofluorescence assay. The medium was removed from the culture wells, and the cell monolayer was rinsed two times with PBS before fixation with pre-chilled methanol-acetic acid (9:1) for 5 min. The wells were rinsed with PBS to remove traces of fixative followed by successive washes with buffer containing 0.1% Triton X-100, 0.35 M NaCl, and 0.13 M Tris-base, pH 7.6 to rehydrate and permeabilize the cells. Normal goat serum (5%) in PBS was used as a blocking solution, and the cell monolayer was stained with a fluorescein-labeled anti-*C*. *parvum* polyclonal antibody (Sporo-Glo; Waterborne Inc.) overnight at 4°C. The stained cells were washed with PBS, followed by rinsing with water, and then imaged with an inverted EVOS M7000 Imaging System (Invitrogen) using a fully automated scan protocol. The EVOS M7000 software was used to program the microscope to autofocus on the center of each well using a 20× objective and then acquire images from multiple fields with overlapping edges to build a single tile-stitched image per well, equating to approximately 40% of the total well area. Celleste Imaging Analysis software (Invitrogen) was used to count the number of intracellular *C. parvum* parasites in each stitched image using the smart segmentation feature to distinguish between objects (parasites) and background (host cells). Experiments were performed in triplicate and repeated three times.

### Synergy analyses

Concentration-response data of each compound mixture and individual compounds obtained from the *in vitro C. parvum* growth inhibition assays were analyzed by multiple approaches to determine the synergistic activity of compound combinations. Dose-response curves were plotted in GraphPad PRISM v8 for curve-shift analysis and calculation of anti-*Cryptosporidium* EC_50_ values by non-linear regression analysis of the mean dose-response curve data normalized to DMSO- and paromomycin-treated wells that represented negative and positive treatment controls, respectively. The combination-index values for the actual experimental data points, as well as the DRI_x_ values at various parasite inhibition levels (*x* = 50%–90%), were derived from the median-effect equation of Chou and Talalay (21) using the CompuSyn software. Compound concentrations for single agents and combinations required to produce a 75% inhibition of the growth of *Cryptosporidium* parasites in cell culture were also calculated by CompuSyn and plotted for isobologram analysis. CI plots and isobolograms were created with GraphPad PRISM v8.

### Testing of the *in vivo* anti-*Cryptosporidium* efficacy of compound combinations

Male IFN-γ KO mice (B6.129S7-*Ifng^tm1Ts^*/J), aged 7 weeks, were procured from The Jackson Laboratory, USA, and housed for 1 week before the start of experiments for acclimatization. Prior to the testing of anti-cryptosporidial efficacy of compounds in mice, groups of mice (*n* = 3 per group) were treated by daily oral gavage with varying dosages of individual compounds and compound combinations for 5 days using a 20 G × 1.5-inch animal feeding needle. During this period, mice were monitored daily for any signs of toxicity including loss of appetite and body weight. Changes in physical and mental activity, body posture, and fur condition were quantified according to a scoring rubric (Table S4). Once non-toxic doses were determined, a newly procured batch of 50 mice was separated into 10 groups (*n* = 5 per group) for efficacy studies. Except for the uninfected control group, all other groups of mice were infected by oral gavage with 5 × 10^4^ *C. parvum* AUCP-1 isolate oocysts suspended in 100 µL of PBS on day 0. Immediately after infection, each individual mouse was housed in a separate cage lined with sterile gauze bedding. On day 3 post infection, infected groups of mice were orally treated with CpPyK (NSC252172 and NSC303244) and CpLDH (NSC158011 and NSC10447) inhibitors either individually or in combination at the indicated doses, 1,000 mg/kg paromomycin in sterile water (positive control), or vehicle (100 µL of 25% DMSO in PEG 400), once daily until day 12. Uninfected control mice were also orally gavaged with the compound vehicle during the treatment period. Mice were weighed daily till study completion. Starting on day 3 after infection, fecal pellets were collected daily in individual sterile 15-mL tubes from each cage and stored at −80°C until use. After treatment discontinuation, two mice from each group were randomly selected and sacrificed on day 13 to assess the effect of compounds on disease development. A small portion (~5 cm) of the distal small intestine just anterior to the cecum was resected from each sacrificed mouse and preserved in 10% neutral buffered formalin for histopathological processing. The remaining mice were maintained up until the termination of study (day 23 post infection) to assess the effect of compound withdrawal on survival and disease relapse/progression. On day 23, all surviving mice were sacrificed, and intestinal tissue samples were collected for histopathological analysis as described above. Harvested intestinal tissues were paraffin embedded and sectioned transversely at a thickness of 5 µm. Sections stained with hematoxylin and eosin were imaged using the EVOS M7000 Imaging System (Invitrogen).

### Quantification of oocyst shedding in fecal samples

Genomic DNA was extracted from 150 mg of feces collected from individual mice by using the QIAamp PowerFecal Pro DNA kit (Qiagen) following the manufacturer’s protocol. Quantification of the oocysts load per gram of feces was performed by qPCR analysis of the Cp18S rRNA gene (GenBank accession number AF164102) using gene-specific primers: 5′-CTGCGAATGGCTCATTATAACA-3′ (Forward) and 5′-AGGCCAATACCCTACCGTCT-3′ (Reverse), described previously (41). To generate quantification standards for qPCR, fecal samples were obtained from uninfected mice and spiked with 10^8^ *C. parvum* oocysts per gram of feces, followed by extraction of DNA as described above. The extracted genomic DNA was then 10-fold serially diluted to generate standard curve quantification standards for qPCR. *C. parvum* oocyst load was quantified for the test mice by using DNA samples from the infected feces in triplicate reactions. Each 20 µL qPCR reaction contained 10 µL of PowerUp SYBR Green Master Mix (Applied Biosystems), 500 nM of each primer, and 2 µL of DNA template. After an initial 5 min denaturation step at 95°C, 40 cycles of denaturation at 95°C for 15 s and annealing/extension at 60°C for 1 min were performed in a QuantStudio 3 Real-Time PCR System (Applied Biosystems). The oocyst load per gram of feces was derived by the QuantStudio 3-system software using the generated quantification standard curves.

### Statistical analyses

All statistical analyses were performed using GraphPad PRISM v8. Normality of data distribution was assessed using Q-Q normal probability plots and the Shapiro-Wilk normality test. Statistical comparisons between the treatment groups and the vehicle control group were done by a non-parametric Kruskal-Wallis test or a parametric one-way analysis of variance test with the Dunn’s or the Dunnett’s multiple comparison *post hoc* tests, respectively, as appropriate for the data. *P* values of 0.05 or less were considered significant. For animal studies, a sample size of five mice per group was found to be sufficient to have a high confidence of detecting a difference in means of at least 25% between treated and untreated groups (two-tailed test using *P* < 0.05) with a power of 90%, as determined by power analyses using the G*Power software (42). This number was consistent with numbers that we have used in the past for similar studies and accounted for animal attrition due to mortality during the study duration.

## SUPPLEMENTAL MATERIAL

Supplemental file 1 aac.00569-23-s0001.pdf Fig. S1 and S2; Tables S1 to S4.

ASM does not own the copyrights to Supplemental Material that may be linked to, or accessed through, an article. The authors have granted ASM a non-exclusive, world-wide license to publish the Supplemental Material files. Please contact the corresponding author directly for reuse.

## References

[1] Zahedi A , Ryan U . 2020. Cryptosporidium - an update with an emphasis on foodborne and waterborne transmission. Res Vet Sci 132:500–512. doi:10.1016/j.rvsc.2020.08.002 32805698

[2] Lima AA , Guerrant DI , Patrick PD , Schorling JB , Moore SR , Guerrant RL . 1999. Association of early childhood diarrhea and cryptosporidiosis with impaired physical fitness and cognitive function four-seven years later in a poor urban community in northeast Brazil. Am J Trop Med Hyg 61:707–713. doi:10.4269/ajtmh.1999.61.707 10586898

[3] Ryan UM , Feng Y , Fayer R , Xiao L . 2021. Taxonomy and molecular epidemiology of Cryptosporidium and Giardia - a 50 year perspective (1971-2021). Int J Parasitol 51:1099–1119. doi:10.1016/j.ijpara.2021.08.007 34715087

[4] Kotloff KL , Nataro JP , Blackwelder WC , Nasrin D , Farag TH , Panchalingam S , Wu Y , Sow SO , Sur D , Breiman RF , Faruque AS , Zaidi AK , Saha D , Alonso PL , Tamboura B , Sanogo D , Onwuchekwa U , Manna B , Ramamurthy T , Kanungo S , Ochieng JB , Omore R , Oundo JO , Hossain A , Das SK , Ahmed S , Qureshi S , Quadri F , Adegbola RA , Antonio M , Hossain MJ , Akinsola A , Mandomando I , Nhampossa T , Acácio S , Biswas K , O’Reilly CE , Mintz ED , Berkeley LY , Muhsen K , Sommerfelt H , Robins-Browne RM , Levine MM . 2013. Burden and aetiology of diarrhoeal disease in infants and young children in developing countries (the global enteric multicenter study, GEMS): a prospective, case-control study. Lancet 382:209–222. doi:10.1016/S0140-6736(13)60844-2 23680352

[5] Platts-Mills JA , Babji S , Bodhidatta L , Gratz J , Haque R , Havt A , McCormick BJ , McGrath M , Olortegui MP , Samie A , Shakoor S , Mondal D , Lima IF , Hariraju D , Rayamajhi BB , Qureshi S , Kabir F , Yori PP , Mufamadi B , Amour C , Carreon JD , Richard SA , Lang D , Bessong P , Mduma E , Ahmed T , Lima AA , Mason CJ , Zaidi AK , Bhutta ZA , Kosek M , Guerrant RL , Gottlieb M , Miller M , Kang G , Houpt ER , Investigators M-E . 2015. Pathogen-specific burdens of community diarrhoea in developing countries: a multisite birth cohort study (MAL-ED). Lancet Glob Health 3:e564–75. doi:10.1016/S2214-109X(15)00151-5 26202075 PMC7328884

[6] Khalil IA , Troeger C , Rao PC , Blacker BF , Brown A , Brewer TG , Colombara DV , De Hostos EL , Engmann C , Guerrant RL , Haque R , Houpt ER , Kang G , Korpe PS , Kotloff KL , Lima AAM , Petri WA , Platts-Mills JA , Shoultz DA , Forouzanfar MH , Hay SI , Reiner RC , Mokdad AH . 2018. Morbidity, mortality, and long-term consequences associated with diarrhoea from Cryptosporidium infection in children younger than 5 years: a meta-analyses study. Lancet Glob Health 6:e758–e768. doi:10.1016/S2214-109X(18)30283-3 29903377 PMC6005120

[7] Nasrin D , Blackwelder WC , Sommerfelt H , Wu Y , Farag TH , Panchalingam S , Biswas K , Saha D , Jahangir Hossain M , Sow SO , Reiman RFB , Sur D , Faruque ASG , Zaidi AKM , Sanogo D , Tamboura B , Onwuchekwa U , Manna B , Ramamurthy T , Kanungo S , Omore R , Ochieng JB , Oundo JO , Das SK , Ahmed S , Qureshi S , Quadri F , Adegbola RA , Antonio M , Mandomando I , Nhampossa T , Bassat Q , Roose A , O’Reilly CE , Mintz ED , Ramakrishnan U , Powell H , Liang Y , Nataro JP , Levine MM , Kotloff KL . 2021. Pathogens associated with linear growth faltering in children with diarrhea and impact of antibiotic treatment: the global enteric multicenter study. J Infect Dis 224:S848–S855. doi:10.1093/infdis/jiab434 34528677 PMC8958895

[8] Desai NT , Sarkar R , Kang G . 2012. Cryptosporidiosis: an under-recognized public health problem. Trop Parasitol 2:91–98. doi:10.4103/2229-5070.105173 23767015 PMC3680871

[9] Wang RJ , Li JQ , Chen YC , Zhang LX , Xiao LH . 2018. Widespread occurrence of Cryptosporidium infections in patients with HIV/AIDS: epidemiology, clinical feature, diagnosis, and therapy. Acta Trop 187:257–263. doi:10.1016/j.actatropica.2018.08.018 30118699

[10] Nsagha DS , Njunda AL , Assob NJC , Ayima CW , Tanue EA , Kibu OD , Kwenti TE . 2016. Intestinal parasitic infections in relation to CD4(+) T cell counts and diarrhea in HIV/AIDS patients with or without antiretroviral therapy in Cameroon. BMC Infect Dis 16:9. doi:10.1186/s12879-016-1337-1 26754404 PMC4707727

[11] Tuli L , Gulati AK , Sundar S , Mohapatra TM . 2008. Correlation between CD4 counts of HIV patients and enteric protozoan in different seasons - an experience of a tertiary care hospital in Varanasi (India). BMC Gastroenterol 8:36. doi:10.1186/1471-230X-8-36 18713475 PMC2536662

[12] Dillingham RA , Pinkerton R , Leger P , Severe P , Guerrant RL , Pape JW , Fitzgerald DW . 2009. High early mortality in patients with chronic acquired immunodeficiency syndrome diarrhea initiating antiretroviral therapy in Haiti: a case-control study. Am J Trop Med Hyg 80:1060–1064. 19478276 PMC3942870

[13] Shaw HJ , Innes EA , Morrison LJ , Katzer F , Wells B . 2020. Long-term production effects of clinical cryptosporidiosis in neonatal calves. Int J Parasitol 50:371–376. doi:10.1016/j.ijpara.2020.03.002 32277986 PMC7194893

[14] Khan SM , Witola WH . 2023. Past, current, and potential treatments for cryptosporidiosis in humans and farm animals: a comprehensive review. Front Cell Infect Microbiol 13:1115522. doi:10.3389/fcimb.2023.1115522 36761902 PMC9902888

[15] Abrahamsen MS , Templeton TJ , Enomoto S , Abrahante JE , Zhu G , Lancto CA , Deng M , Liu C , Widmer G , Tzipori S , Buck GA , Xu P , Bankier AT , Dear PH , Konfortov BA , Spriggs HF , Iyer L , Anantharaman V , Aravind L , Kapur V . 2004. Complete genome sequence of the apicomplexan, Cryptosporidium parvum. Science 304:441–445. doi:10.1126/science.1094786 15044751

[16] Cook WJ , Senkovich O , Aleem K , Chattopadhyay D . 2012. Crystal structure of Cryptosporidium parvum pyruvate kinase. PLoS One 7:e46875. doi:10.1371/journal.pone.0046875 23056503 PMC3467265

[17] Cook WJ , Senkovich O , Hernandez A , Speed H , Chattopadhyay D . 2015. Biochemical and structural characterization of Cryptosporidium parvum lactate dehydrogenase. Int J Biol Macromol 74:608–619. doi:10.1016/j.ijbiomac.2014.12.019 25542170

[18] Li K , Nader SM , Zhang X , Ray BC , Kim CY , Das A , Witola WH . 2019. Novel lactate dehydrogenase inhibitors with in vivo efficacy against Cryptosporidium parvum. PLoS Pathog 15:e1007953. doi:10.1371/journal.ppat.1007953 31356619 PMC6687188

[19] Khan SM , Zhang X , Witola WH . 2022. Cryptosporidium parvum pyruvate kinase inhibitors with in vivo anti-cryptosporidial efficacy. Front Microbiol 12:800293. doi:10.3389/fmicb.2021.800293 35046922 PMC8761912

[20] Straetemans R , O’Brien T , Wouters L , Van Dun J , Janicot M , Bijnens L , Burzykowski T , Aerts M . 2005. Design and analysis of drug combination experiments. Biom J 47:299–308. doi:10.1002/bimj.200410124 16053254

[21] Chou TC , Talalay P . 1984. Quantitative analysis of dose-effect relationships: the combined effects of multiple drugs or enzyme inhibitors. Adv Enzyme Regul 22:27–55. doi:10.1016/0065-2571(84)90007-4 6382953

[22] Griffiths JK , Theodos C , Paris M , Tzipori S . 1998. The gamma interferon gene knockout mouse: a highly sensitive model for evaluation of therapeutic agents against Cryptosporidium parvum. J Clin Microbiol 36:2503–2508. doi:10.1128/JCM.36.9.2503-2508.1998 9705383 PMC105153

[23] Checkley W , White AC , Jaganath D , Arrowood MJ , Chalmers RM , Chen X-M , Fayer R , Griffiths JK , Guerrant RL , Hedstrom L , Huston CD , Kotloff KL , Kang G , Mead JR , Miller M , Petri WA , Priest JW , Roos DS , Striepen B , Thompson RCA , Ward HD , Van Voorhis WA , Xiao L , Zhu G , Houpt ER . 2015. A review of the global burden, novel diagnostics, therapeutics, and vaccine targets for cryptosporidium. Lancet Infect Dis 15:85–94. doi:10.1016/S1473-3099(14)70772-8 25278220 PMC4401121

[24] Santin M . 2020. Cryptosporidium and Giardia in ruminants. Vet Clin North Am Food Anim Pract 36:223–238. doi:10.1016/j.cvfa.2019.11.005 32029186

[25] Lanternier F , Amazzough K , Favennec L , Mamzer-Bruneel M-F , Abdoul H , Tourret J , Decramer S , Zuber J , Scemla A , Legendre C , Lortholary O , Bougnoux M-E , ANOFEL Cryptosporidium National Network and Transplant Cryptosporidium Study Group . 2017. Cryptosporidium Spp. infection in solid organ transplantation: the nationwide "TRANSCRYPTO" study. Transplantation 101:826–830. doi:10.1097/TP.0000000000001503 27681270 PMC7228595

[26] Maggi P , Larocca AM , Quarto M , Serio G , Brandonisio O , Angarano G , Pastore G . 2000. Effect of antiretroviral therapy on cryptosporidiosis and microsporidiosis in patients infected with human immunodeficiency virus type 1. Eur J Clin Microbiol Infect Dis 19:213–217. doi:10.1007/s100960050461 10795595

[27] Love MS , Choy RKM . 2021. Emerging treatment options for cryptosporidiosis. Curr Opin Infect Dis 34:455–462. doi:10.1097/QCO.0000000000000761 34261904 PMC7611666

[28] Giacometti A , Cirioni O , Barchiesi F , Ancarani F , Scalise G . 2000. Activity of Nitazoxanide alone and in combination with azithromycin and rifabutin against Cryptosporidium parvum in cell culture. J Antimicrob Chemother 45:453–456. doi:10.1093/jac/45.4.453 10747821

[29] Hommer V , Eichholz J , Petry F . 2003. Effect of antiretroviral protease inhibitors alone, and in combination with paromomycin, on the excystation, invasion and in vitro development of Cryptosporidium parvum. J Antimicrob Chemother 52:359–364. doi:10.1093/jac/dkg357 12888587

[30] You X , Schinazi RF , Arrowood MJ , Lejkowski M , Juodawlkis AS , Mead JR . 1998. In vitro activities of paromomycin and lasalocid evaluated in combination against Cryptosporidium parvum. J Antimicrob Chemother 41:293–296. doi:10.1093/jac/41.2.293 9533476

[31] Mauzy MJ , Enomoto S , Lancto CA , Abrahamsen MS , Rutherford MS . 2012. The Cryptosporidium parvum transcriptome during in vitro development. PLoS One 7:e31715. doi:10.1371/journal.pone.0031715 22438867 PMC3305300

[32] Tandel J , English ED , Sateriale A , Gullicksrud JA , Beiting DP , Sullivan MC , Pinkston B , Striepen B . 2019. Life cycle progression and sexual development of the apicomplexan parasite Cryptosporidium parvum. Nat Microbiol 4:2226–2236. doi:10.1038/s41564-019-0539-x 31477896 PMC6877471

[33] Mirhashemi ME , Noubary F , Chapman-Bonofiglio S , Tzipori S , Huggins GS , Widmer G . 2018. Transcriptome analysis of pig intestinal cell monolayers infected with Cryptosporidium parvum asexual stages. Parasit Vectors 11:176. doi:10.1186/s13071-018-2754-3 29530089 PMC5848449

[34] Zhang H , Guo F , Zhu G , Knoll LJ . 2015. Cryptosporidium lactate dehydrogenase is associated with the parasitophorous vacuole membrane and is a potential target for developing therapeutics. PLoS Pathog 11:e1005250. doi:10.1371/journal.ppat.1005250 26562790 PMC4642935

[35] Hasan MM , Stebbins EE , Choy RKM , Gillespie JR , de Hostos EL , Miller P , Mushtaq A , Ranade RM , Teixeira JE , Verlinde C , Sateriale A , Zhang Z , Osbourn DM , Griggs DW , Fan E , Buckner FS , Huston CD . 2021. Spontaneous selection of Cryptosporidium drug resistance in a calf model of infection. Antimicrob Agents Chemother 65:e00023-21. doi:10.1128/AAC.00023-21 33753338 PMC8316126

[36] Tallarida RJ . 2011. Quantitative methods for assessing drug synergism. Genes Cancer 2:1003–1008. doi:10.1177/1947601912440575 22737266 PMC3379564

[37] Arrowood MJ , Sterling CR . 1987. Isolation of Cryptosporidium oocysts and sporozoites using discontinuous sucrose and isopycnic percoll gradients. J Parasitol 73:314–319. 3585626

[38] Current WL . 1990. Techniques and laboratory maintenance of *Cryptosporidium* , p 44–77. In Dubey JP , CA Speer , R Fayer (ed), Cryptosporidiosis of man and animals. CRC Press, Boca Raton, Fla.

[39] Kuhlenschmidt TB , Rutaganira FU , Long S , Tang K , Shokat KM , Kuhlenschmidt MS , Sibley LD . 2016. Inhibition of calcium-dependent protein kinase 1 (CDPK1) in vitro by pyrazolopyrimidine derivatives does not correlate with sensitivity of Cryptosporidium parvum growth in cell culture. Antimicrob Agents Chemother 60:570–579. doi:10.1128/AAC.01915-15 26552986 PMC4704151

[40] Khan SM , Garcia Hernandez A , Allaie IM , Grooms GM , Li K , Witola WH , Stec J . 2022. Activity of (1-benzyl-4-triazolyl)-indole-2-carboxamides against Toxoplasma gondii and Cryptosporidium parvum. Int J Parasitol Drugs Drug Resist 19:6–20. doi:10.1016/j.ijpddr.2022.04.001 35462232 PMC9046076

[41] Parr JB , Sevilleja JE , Samie A , Alcantara C , Stroup SE , Kohli A , Fayer R , Lima AAM , Houpt ER , Guerrant RL . 2007. Detection and quantification of Cryptosporidium in HCT-8 cells and human fecal specimens using real-time polymerase chain reaction. Am J Trop Med Hyg 76:938–942. 17488919 PMC2253489

[42] Faul F , Erdfelder E , Lang AG , Buchner A . 2007. G*Power 3: a flexible statistical power analysis program for the social, behavioral, and biomedical sciences. Behav Res Methods 39:175–191. doi:10.3758/bf03193146 17695343

