# Supplementary material for: Combination of inhibitors for two glycolytic enzymes portrays high synergistic efficacy against Cryptosporidium parvum
Source: Antimicrob Agents Chemother. 2023 Sep 1;67(10):e00569-23. doi: 10.1128/aac.00569-23 (PMC10583678; doi:10.1128/aac.00569-23)
Supplement: Supplemental file 1 — Fig. S1 and S2; Tables S1 to S4. [file aac.00569-23-s0001.pdf]

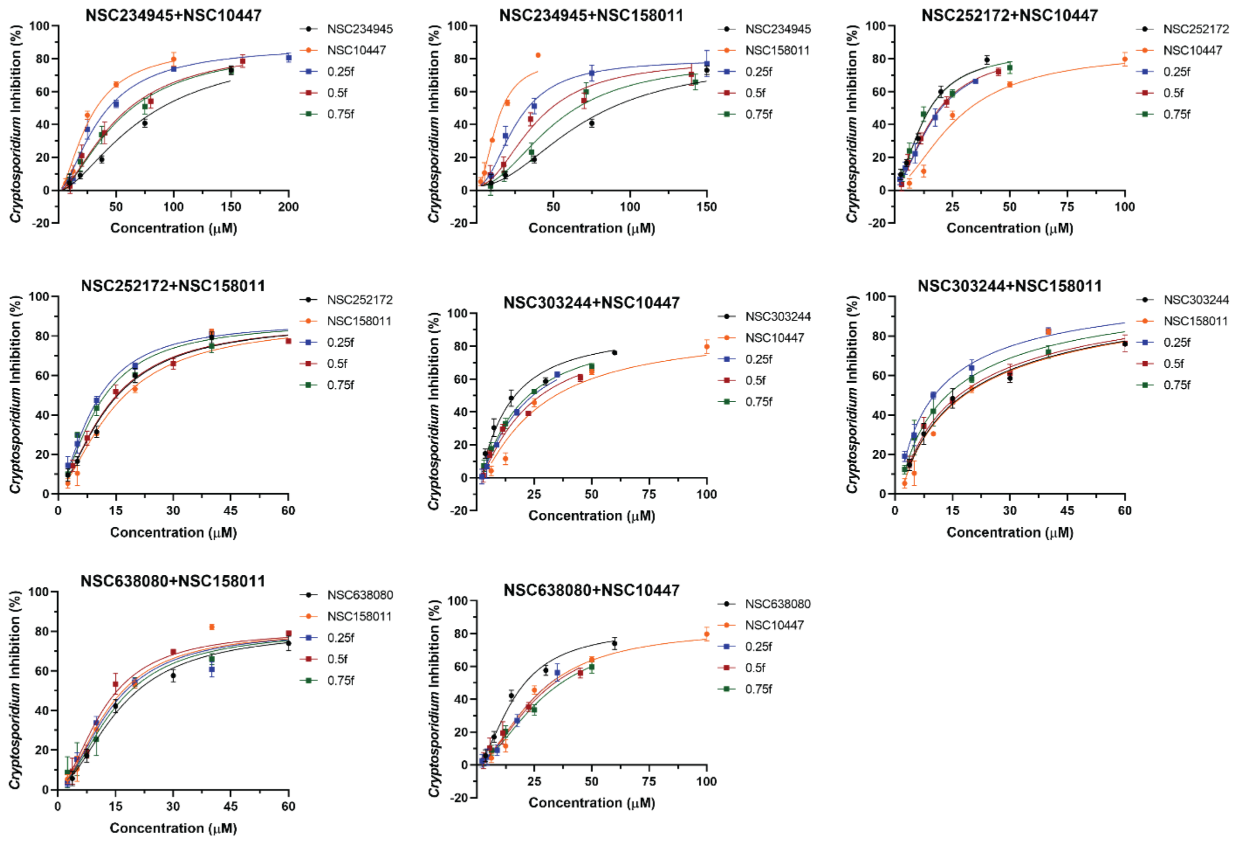

FIG S1 Curve-shift analyses of individual CpPyK- and CpLDH-inhibitors and their combinations at different combination ratios. CpPyK- and CpLDH-inhibitors were combined in concentration ratios determined by 3 values of mixture factor (*f*) to prepare 3 different compound combinations (0.25*f*, 0.5*f*, and 0.75*f*). Concentration-response data of each compound mixture and individual compounds acquired from the *in vitro* parasite growth inhibition assays were plotted in GraphPad PRISM® v8. A leftward shift of curves for combinations compared with individual compound curves indicates synergism and a rightward shift indicates antagonism. Data are presented as mean ± standard deviation of the mean (SD) of three independent experiments.

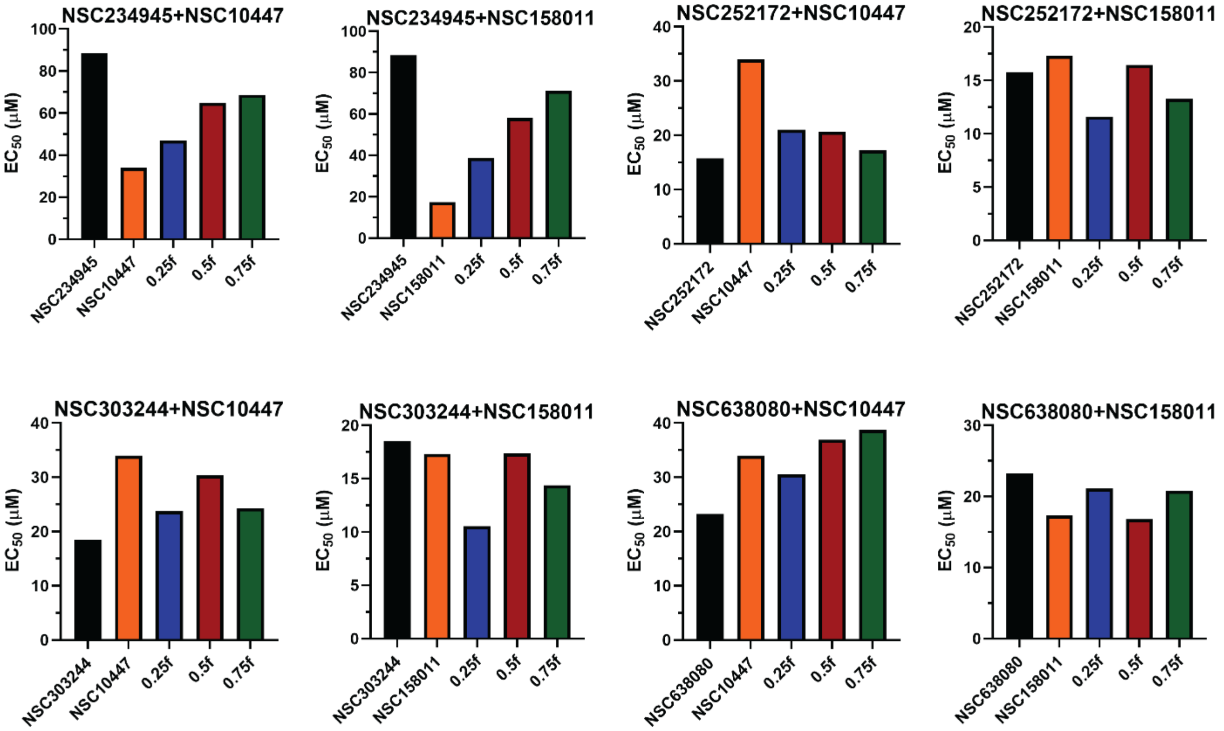

FIG S2 Comparison of anti-*Cryptosporidium* EC_{50} values of individual CpPyK- and CpLDH-inhibitors and their combinations at different combination ratios. CpPyK- and CpLDH-inhibitors were combined in concentration ratios determined by 3 values of mixture factor (*f*) to prepare 3 different combinations (0.25*f*, 0.5*f*, and 0.75*f*). Concentration-response data points of each compound mixture and individual compounds obtained from the *in vitro C. parvum* growth inhibition assays were analyzed by the non-linear regression equation in GraphPad PRISM® v8 software to calculate EC_{50} values. The EC_{50} of the individual drugs were compared to the resulting EC_{50} after combination treatments. The data shown represent the mean EC_{50} values of three independent experiments.

TABLE S1 Mean percent cytotoxicity of combinations of CpPyK- and CpLDH-inhibitors in HCT-8 cells.

0.25*f*							
Conc. (µM)		Mean Percent Cytotoxicity (%)					
NSC234945	NSC10447	1	2	3	AVG	STDEV	SEM
0	0	0.4	4.5	-4.9	**0.0**	4.7	2.7
12.5	12.5	-0.4	1.4	1.5	**0.8**	1.1	0.6
25	25	10.5	-0.6	7.1	**5.7**	5.7	3.3
50	50	10.9	11.8	1.6	**8.1**	5.6	3.2
100	100	17.0	19.4	23.4	**19.9**	3.3	1.9
200	200	40.7	40.5	46.1	**42.4**	3.2	1.8

0.5*f*							
Conc. (µM)		Mean Percent Cytotoxicity (%)					
NSC234945	NSC10447	1	2	3	AVG	STDEV	SEM
0	0	-0.5	0.7	-0.1	**0.0**	0.6	0.4
15	5	5.6	-5.1	3.3	**1.2**	5.6	3.3
30	10	4.9	9.8	3.2	**6.0**	3.4	2.0
60	20	5.1	9.6	15.4	**10.0**	5.2	3.0
120	40	18.6	21.5	13.4	**17.8**	4.1	2.4
240	80	24.1	37.8	28.5	**30.2**	7.0	4.0

0.75*f*							
Conc. (µM)		Mean Percent Cytotoxicity (%)					
NSC234945	NSC10447	1	2	3	AVG	STDEV	SEM
0	0	-1.4	5.6	-4.2	**0.0**	5.1	2.9
16.875	1.875	2.1	-1.2	5.2	**2.1**	3.2	1.8
33.75	3.75	6.3	4.4	2.5	**4.4**	1.9	1.1
67.5	7.5	8.4	0.5	11.3	**6.7**	5.6	3.2
135	15	15.3	11.4	5.9	**10.9**	4.7	2.7
270	30	37.4	21.1	33.6	**30.7**	8.5	4.9

0.25*f*							
Conc. (µM)		Mean Percent Cytotoxicity (%)					
NSC234945	NSC158011	1	2	3	AVG	STDEV	SEM
0	0	-5.7	2.6	3.1	**0.0**	4.9	2.9
12.5	6.25	-5.1	0.1	2.3	**-0.9**	3.8	2.2
25	12.5	1.6	6.0	0.1	**2.6**	3.1	1.8
50	25	3.7	16.5	8.8	**9.6**	6.5	3.7
100	50	17.7	27.6	13.5	**19.6**	7.2	4.2
200	100	34.7	40.9	34.0	**36.6**	3.8	2.2

0.5*f*							
Conc. (µM)		Mean Percent Cytotoxicity (%)					
NSC234945	NSC158011	1	2	3	AVG	STDEV	SEM
0	0	-5.0	5.7	-0.7	**0.0**	5.4	3.1
15	2.5	-2.4	8.1	1.0	**2.2**	5.3	3.1
30	5	4.8	6.5	0.4	**3.9**	3.1	1.8
60	10	11.3	5.9	12.8	**10.0**	3.6	2.1
120	20	21.2	12.6	17.0	**16.9**	4.3	2.5
240	40	36.4	33.7	27.6	**32.6**	4.5	2.6

0.75*f*							
Conc. (µM)		Mean Percent Cytotoxicity (%)					
NSC234945	NSC158011	1	2	3	AVG	STDEV	SEM
0	0	2.8	2.2	-5.1	**0.0**	4.4	2.5
16.875	0.9375	4.6	3.7	-5.7	**0.9**	5.7	3.3
33.75	1.875	7.6	4.2	1.6	**4.5**	3.0	1.7
67.5	3.75	5.3	6.3	10.0	**7.2**	2.5	1.5
135	7.5	10.5	17.8	6.7	**11.6**	5.6	3.2
270	15	29.6	25.3	21.0	**25.3**	4.3	2.5

0.25*f*							
Conc. (µM)		Mean Percent Cytotoxicity (%)					
NSC252172	NSC10447	1	2	3	AVG	STDEV	SEM
0	0	4.7	-3.5	-1.2	**0.0**	4.2	2.4
1.25	7.5	1.1	13.8	4.9	**6.6**	6.5	3.8
2.5	15	12.8	12.1	16.9	**13.9**	2.6	1.5
5	30	20.4	14.0	23.6	**19.3**	4.9	2.8
10	60	28.5	22.9	34.3	**28.6**	5.7	3.3
20	120	54.4	47.4	49.7	**50.5**	3.6	2.1

0.5*f*							
Conc. (µM)		Mean Percent Cytotoxicity (%)					
NSC252172	NSC10447	1	2	3	AVG	STDEV	SEM
0	0	-5.2	-2.8	8.0	**0.0**	7.0	4.1
3.75	7.5	13.0	2.7	-2.8	**4.3**	8.0	4.6
7.5	15	13.3	16.0	4.2	**11.2**	6.2	3.6
15	30	14.6	24.3	20.4	**19.8**	4.9	2.8
30	60	29.8	27.0	38.2	**31.7**	5.8	3.4
60	120	63.0	57.3	60.1	**60.1**	2.8	1.6

0.75*f*							
Conc. (µM)		Mean Percent Cytotoxicity (%)					
NSC252172	NSC10447	1	2	3	AVG	STDEV	SEM
0	0	8.8	-8.3	-0.5	**0.0**	8.5	4.9
7.5	5	12.8	8.1	-3.9	**5.7**	8.6	5.0
15	10	13.0	17.0	2.1	**10.7**	7.7	4.5
30	20	18.7	13.7	23.3	**18.6**	4.8	2.8
60	40	40.6	34.5	27.3	**34.1**	6.7	3.9
120	80	54.0	59.6	60.3	**58.0**	3.4	2.0

0.25*f*							
Conc. (µM)		Mean Percent Cytotoxicity (%)					
NSC252172	NSC158011	1	2	3	AVG	STDEV	SEM
0	0	2.2	0.8	-3.0	**0.0**	2.7	1.5
2.5	7.5	6.1	5.0	-1.0	**3.4**	3.8	2.2
5	15	8.5	14.2	6.9	**9.9**	3.8	2.2
10	30	23.1	12.4	14.6	**16.7**	5.7	3.3
20	60	36.4	30.1	26.0	**30.8**	5.3	3.0
40	120	48.6	39.9	40.5	**43.0**	4.9	2.8

0.5*f*							
Conc. (µM)		Mean Percent Cytotoxicity (%)					
NSC252172	NSC158011	1	2	3	AVG	STDEV	SEM
0	0	1.2	5.0	-6.2	**0.0**	5.7	3.3
7.5	7.5	-2.1	2.7	6.5	**2.4**	4.3	2.5
15	15	16.5	6.2	11.9	**11.6**	5.1	3.0
30	30	25.6	12.8	19.3	**19.2**	6.4	3.7
60	60	38.6	43.7	50.9	**44.4**	6.2	3.6
120	120	61.2	63.1	56.0	**60.1**	3.7	2.1

0.75*f*							
Conc. (µM)		Mean Percent Cytotoxicity (%)					
NSC252172	NSC158011	1	2	3	AVG	STDEV	SEM
0	0	-0.7	0.5	0.2	**0.0**	0.6	0.3
7.5	2.5	-2.4	6.5	3.5	**2.6**	4.5	2.6
15	5	6.5	15.1	4.9	**8.8**	5.5	3.2
30	10	14.3	16.7	24.4	**18.5**	5.3	3.0
60	20	33.1	42.2	34.6	**36.6**	4.9	2.8
120	40	57.6	56.1	51.5	**55.1**	3.2	1.9

0.25*f*							
Conc. (µM)		Mean Percent Cytotoxicity (%)					
NSC303244	NSC10447	1	2	3	AVG	STDEV	SEM
0	0	-10.4	7.0	3.4	**0.0**	9.2	5.3
1.25	7.5	-0.2	-6.5	11.6	**1.6**	9.2	5.3
2.5	15	-1.2	8.3	12.0	**6.4**	6.8	3.9
5	30	19.1	3.9	11.4	**11.5**	7.6	4.4
10	60	18.9	22.1	29.0	**23.4**	5.2	3.0
20	120	37.3	43.5	36.9	**39.2**	3.7	2.1

0.5*f*							
Conc. (µM)		Mean Percent Cytotoxicity (%)					
NSC303244	NSC10447	1	2	3	AVG	STDEV	SEM
0	0	-8.4	1.9	6.5	**0.0**	7.6	4.4
3.75	7.5	8.5	8.3	-1.7	**5.0**	5.8	3.4
7.5	15	-0.4	14.4	11.4	**8.4**	7.8	4.5
15	30	22.5	20.0	6.1	**16.2**	8.8	5.1
30	60	27.9	23.1	33.0	**28.0**	5.0	2.9
60	120	45.0	54.8	47.4	**49.1**	5.1	3.0

0.75*f*							
Conc. (µM)		Mean Percent Cytotoxicity (%)					
NSC303244	NSC10447	1	2	3	AVG	STDEV	SEM
0	0	6.9	-2.1	-4.8	**0.0**	6.1	3.5
7.5	5	0.0	-3.5	11.1	**2.5**	7.7	4.4
15	10	11.2	6.3	1.5	**6.3**	4.9	2.8
30	20	21.7	12.4	17.6	**17.2**	4.7	2.7
60	40	22.0	27.9	24.7	**24.9**	3.0	1.7
120	80	34.1	42.6	41.4	**39.4**	4.6	2.6

**0.25*f***							
**Conc. (µM)**		**Mean Percent Cytotoxicity (%)**					
**NSC303244**	**NSC158011**	**1**	**2**	**3**	**AVG**	**STDEV**	**SEM**
0	0	-1.9	-0.3	2.2	**0.0**	2.1	1.2
2.5	7.5	-4.0	7.7	5.9	**3.2**	6.3	3.7
5	15	3.4	6.9	1.2	**3.8**	2.9	1.7
10	30	5.6	13.8	9.5	**9.6**	4.1	2.4
20	60	25.7	21.2	16.4	**21.1**	4.6	2.7
40	120	27.0	34.6	26.4	**29.4**	4.6	2.7

**0.5*f***							
**Conc. (µM)**		**Mean Percent Cytotoxicity (%)**					
**NSC303244**	**NSC158011**	**1**	**2**	**3**	**AVG**	**STDEV**	**SEM**
0	0	0.3	-1.1	0.8	**0.0**	1.0	0.6
7.5	7.5	6.7	9.0	2.1	**5.9**	3.5	2.0
15	15	7.6	16.3	9.6	**11.1**	4.6	2.6
30	30	10.2	18.6	18.4	**15.7**	4.8	2.8
60	60	34.1	31.8	39.9	**35.3**	4.2	2.4
120	120	45.7	48.9	53.6	**49.4**	4.0	2.3

**0.75*f***							
**Conc. (µM)**		**Mean Percent Cytotoxicity (%)**					
**NSC303244**	**NSC158011**	**1**	**2**	**3**	**AVG**	**STDEV**	**SEM**
0	0	3.7	1.0	-4.7	**0.0**	4.3	2.5
7.5	2.5	9.1	3.7	-0.8	**4.0**	5.0	2.9
15	5	11.3	7.6	-0.4	**6.2**	6.0	3.4
30	10	15.2	13.7	6.0	**11.6**	4.9	2.9
60	20	19.2	20.0	27.7	**22.3**	4.7	2.7
120	40	29.3	34.1	39.2	**34.2**	4.9	2.9

**0.25*f***							
**Conc. (µM)**		**Mean Percent Cytotoxicity (%)**					
**NSC638080**	**NSC10447**	**1**	**2**	**3**	**AVG**	**STDEV**	**SEM**
0	0	-5.0	6.8	-1.8	**0.0**	6.1	3.5
1.25	7.5	2.8	10.3	-1.1	**4.0**	5.8	3.3
2.5	15	1.2	9.9	11.5	**7.5**	5.6	3.2
5	30	14.5	12.4	20.9	**15.9**	4.4	2.6
10	60	27.4	27.4	24.9	**26.5**	1.4	0.8
20	120	43.7	49.6	42.2	**45.1**	3.9	2.3

**0.5*f***							
**Conc. (µM)**		**Mean Percent Cytotoxicity (%)**					
**NSC638080**	**NSC10447**	**1**	**2**	**3**	**AVG**	**STDEV**	**SEM**
0	0	-2.5	-2.6	5.1	**0.0**	4.5	2.6
3.75	7.5	0.1	7.6	9.2	**5.6**	4.9	2.8
7.5	15	10.1	8.4	15.0	**11.2**	3.5	2.0
15	30	13.1	20.7	21.9	**18.5**	4.8	2.8
30	60	28.0	26.6	35.4	**30.0**	4.7	2.7
60	120	48.4	46.0	52.9	**49.1**	3.5	2.0

**0.75*f***							
**Conc. (µM)**		**Mean Percent Cytotoxicity (%)**					
**NSC638080**	**NSC10447**	**1**	**2**	**3**	**AVG**	**STDEV**	**SEM**
0	0	5.7	-5.0	-0.7	**0.0**	5.4	3.1
7.5	5	2.5	-2.3	4.8	**1.7**	3.6	2.1
15	10	6.2	-0.6	9.4	**5.0**	5.1	2.9
30	20	24.4	17.3	16.9	**19.5**	4.2	2.4
60	40	21.7	24.7	27.8	**24.8**	3.1	1.8
120	80	39.7	39.0	38.5	**39.1**	0.6	0.3

**0.25*f***							
**Conc. (µM)**		**Mean Percent Cytotoxicity (%)**					
**NSC638080**	**NSC158011**	**1**	**2**	**3**	**AVG**	**STDEV**	**SEM**
0	0	-3.2	3.3	-0.1	**0.0**	3.3	1.9
2.5	7.5	1.1	0.2	8.6	**3.3**	4.6	2.7
5	15	14.3	2.5	9.4	**8.7**	5.9	3.4
10	30	20.8	10.3	5.8	**12.3**	7.7	4.4
20	60	18.9	22.4	28.4	**23.2**	4.8	2.8
40	120	29.3	34.5	40.4	**34.7**	5.6	3.2

**0.5*f***							
**Conc. (µM)**		**Mean Percent Cytotoxicity (%)**					
**NSC638080**	**NSC158011**	**1**	**2**	**3**	**AVG**	**STDEV**	**SEM**
0	0	-2.5	2.5	-0.1	**0.0**	2.5	1.4
7.5	7.5	1.3	7.4	1.9	**3.5**	3.4	1.9
15	15	12.8	11.0	21.0	**14.9**	5.3	3.1
30	30	16.0	24.6	19.0	**19.9**	4.4	2.5
60	60	45.7	42.8	45.4	**44.6**	1.6	0.9
120	120	60.5	60.3	55.7	**58.9**	2.7	1.6

**0.75*f***							
**Conc. (µM)**		**Mean Percent Cytotoxicity (%)**					
**NSC638080**	**NSC158011**	**1**	**2**	**3**	**AVG**	**STDEV**	**SEM**
0	0	-3.2	-0.1	3.3	**0.0**	3.2	1.9
7.5	2.5	-5.1	6.0	3.0	**1.3**	5.7	3.3
15	5	2.9	5.2	13.0	**7.0**	5.3	3.0
30	10	12.1	16.4	14.6	**14.4**	2.1	1.2
60	20	25.2	26.8	14.7	**22.2**	6.6	3.8
120	40	33.2	35.3	38.8	**35.8**	2.8	1.6

TABLE S2 Dose-reduction index (DRI) values of compound mixtures at the parasite inhibition effective concentration (EC) of 50%, 75%, and 90% (EC_{50}, EC_{75}, and EC_{90}, respectively).

**Compound mixture (CpPyKi + CpLDHi)**	**Mixture factor (*f*)**	**DRI value of CpPyKi and CpLDHi at**					
		**EC_{50}**		**EC_{75}**		**EC_{90}**	
		**CpPyKi**	**CpLDHi**	**CpPyKi**	**CpLDHi**	**CpPyKi**	**CpLDHi**
NSC234945 + NSC10447	0.25	3.29	1.39	1.79	2.27	1.44	5.47
	0.50	3.15	1.20	1.95	2.24	1.30	4.47
	0.75	3.01	1.04	2.13	2.20	1.17	3.64
NSC234945 + NSC158011	0.25	3.06	1.17	1.71	1.96	1.25	4.30
	0.50	2.74	0.98	1.44	1.53	1.35	4.31
	0.75	2.45	0.81	1.20	1.20	1.45	4.32
NSC252172 + NSC10447	0.25	5.09	2.05	2.22	2.69	1.43	5.20
	0.50	4.64	1.57	2.42	2.46	1.31	4.00
	0.75	4.22	1.21	2.63	2.25	1.20	3.08
NSC252172 + NSC158011	0.25	5.31	1.93	1.80	1.96	1.50	4.91
	0.50	4.77	1.51	1.52	1.44	1.30	3.69
	0.75	4.28	1.17	1.28	1.05	1.12	2.77
NSC303244 + NSC10447	0.25	5.96	1.98	2.06	2.05	1.28	3.82
	0.50	5.03	2.15	3.01	1.94	1.51	2.91
	0.75	4.87	2.35	3.39	1.83	1.77	2.22
NSC303244 + NSC158011	0.25	7.29	2.18	2.14	1.91	1.72	4.62
	0.50	7.77	1.55	2.05	1.23	1.73	3.12
	0.75	8.28	1.11	1.96	0.79	1.75	2.11
NSC638080 + NSC10447	0.25	4.96	1.27	2.10	1.61	1.08	2.50
	0.50	5.05	1.13	2.03	1.36	1.07	2.15
	0.75	5.14	1.00	1.97	1.15	1.06	1.86
NSC638080 + NSC158011	0.25	4.60	1.06	2.64	1.83	1.53	3.17
	0.50	4.55	0.95	2.61	1.63	1.28	2.40
	0.75	4.50	0.84	2.59	1.45	1.08	1.82

TABLE S3 Effect of oral administration of CpPyK- and CpLDH-inhibitors on the physical indicators of health in mice.

Treatment	Dose (mg/kg)	Day	Average Body Weight (g)	Average Score*			
				Activity	Skin/ Fur	Posture	Mental State
NSC252172	50	1	23.7	4	4	4	4
		2	24.4	4	4	4	4
		3	24.7	4	4	4	4
		4	24.9	4	4	4	4
		5	24.8	4	4	4	4
	75	1	24.4	4	4	4	4
		2	24.6	4	4	4	4
		3	24.5	4	4	4	4
		4	24.7	4	4	4	4
		5	24.9	4	4	4	4
	100	1	24.6	4	4	4	4
		2	24.6	4	4	4	4
		3	25.1	4	4	4	4
		4	25.1	4	4	4	4
		5	25.3	4	4	4	4
NSC303244	50	1	21.9	4	4	4	4
		2	21.6	4	4	4	4
		3	22.2	4	4	4	4
		4	22.4	4	4	4	4
		5	22.4	4	4	4	4
	75	1	23.7	4	4	4	4
		2	23.6	4	4	4	4
		3	23.5	4	4	4	4
		4	23.2	4	4	4	4
		5	22.7	3	3	4	4
	100	1	23.1	4	4	4	4
		2	22.9	4	4	4	4
		3	22.7	4	4	4	4
		4	22	3	3	4	3
		5	20.9	3	2	3	3
NSC158011	75	1	22	4	4	4	4
		2	22.2	4	4	4	4
		3	22.2	4	4	4	4
		4	22.1	4	4	4	4
		5	22.4	4	4	4	4
	150	1	22.4	4	4	4	4
		2	22.6	4	4	4	4
		3	22.8	4	4	4	4
		4	22.9	4	4	4	4
		5	23.4	4	4	4	4
	200	1	22.9	4	4	4	4
		2	22.7	4	4	4	4
		3	22.9	4	4	4	4
		4	23.2	4	4	4	4
		5	23.3	4	4	4	4
NSC10447	100	1	22.7	4	4	4	4
		2	22.9	4	4	4	4

		3	22.8	4	4	4	4
		4	22.9	4	4	4	4
		5	23	4	4	4	4
	200	1	24.4	4	4	4	4
		2	24.9	4	4	4	4
		3	24.9	4	4	4	4
		4	25	4	4	4	4
		5	25.3	4	4	4	4
	400	1	22.6	4	4	4	4
		2	23	4	4	4	4
		3	23.2	4	4	4	4
		4	23	4	4	4	4
		5	23	4	4	4	4
NSC252172 + NSC10447 (1:2)	75	1	22.9	4	4	4	4
		2	23.3	4	4	4	4
		3	23.3	4	4	4	4
		4	23.5	4	4	4	4
		5	23.9	4	4	4	4
	150	1	24.6	4	4	4	4
		2	24.5	4	4	4	4
		3	24.4	4	4	4	4
		4	24.5	4	4	4	4
		5	24.4	4	4	4	4
	300	1	22.4	4	4	4	4
		2	22.7	4	4	4	4
		3	22.5	4	4	4	4
		4	22.6	4	4	4	4
		5	22.7	4	4	4	4
NSC252172 + NSC158011 (1:3)	50	1	23.9	4	4	4	4
		2	23.9	4	4	4	4
		3	24.1	4	4	4	4
		4	23.9	4	4	4	4
		5	24	4	4	4	4
	100	1	24.7	4	4	4	4
		2	24.9	4	4	4	4
		3	24.7	4	4	4	4
		4	25	4	4	4	4
		5	24.9	4	4	4	4
	200	1	23.5	4	4	4	4
		2	23.8	4	4	4	4
		3	23.7	4	4	4	4
		4	23.9	4	4	4	4
		5	24.1	4	4	4	4
NSC303244 + NSC158011 (1:3)	50	1	25.1	4	4	4	4
		2	25.4	4	4	4	4
		3	25.6	4	4	4	4
		4	25.5	4	4	4	4
		5	25.5	4	4	4	4
	100	1	23.4	4	4	4	4
		2	23.6	4	4	4	4
		3	23.8	4	4	4	4
		4	23.7	4	4	4	4
		5	23.9	4	4	4	4

200	1	24	4	4	4	4
	2	23.8	4	4	4	4
	3	24	4	4	4	4
	4	23.9	4	4	4	4
	5	23.9	4	4	4	4

* See Table S4 for the scoring rubric.

TABLE S4 Criteria used for scoring changes in physical activity, fur condition, body posture, and mental state due to compound toxicity in mice.

**Score**	**Physical activity**	**Skin/ Fur**	**Posture**	**Mental State**
1	immobile	sparse	lying flat	lifeless/ trembling
2	bradykinesia	rough/ lackluster	hunched	depressed
3	slightly sluggish	little rough/ lackluster	slightly hunched	agitated
4	fully active	smooth/ glossy	normal standing	good spirit
